# Transcriptome analysis reveals biosynthesis and regulation of flavonoid in common bean seeds during grain filling

**DOI:** 10.1186/s12870-024-05593-5

**Published:** 2024-10-01

**Authors:** Gerardo Tapia, Máximo Gonzalez, José Méndez, Guillermo Schmeda-Hirschmann, Oscar Arrey, Basilio Carrasco, Nélida Nina, Alexis Salas-Burgos, Felipe Jimenéz-Aspee, Barbara Arevalo

**Affiliations:** 1 Unidad de Recursos Genéticos Vegetales, Instituto de Investigaciones Agropecuarias, INIA- Quilamapu, Chillán, 3800062 Chile; 2 Laboratorio de Fisiología Vegetal, Centro de Estudios Avanzados en Zonas Áridas (CEAZA), Raúl Bitrán 1305, La Serena, Chile; 3 Laboratorio de Química de Productos Naturales, Instituto de Química de Recursos Naturales, Universidad de Talca, Talca, 3480094 Chile; 4 Centro de Estudios en Alimentos Procesados (CEAP), Campus Lircay, Talca, 3480094 Chile; 5 Departamento de Farmacología, Facultad de Ciencias Biológicas, Universidad de Concepción, Concepción, 4070386 Chile; 6 Department of Food Biofunctionality, Institute of Nutritional Sciences, University of Hohenheim, 70599 Stuttgart, Germany

**Keywords:** Anthocyanin, Common bean, Seed development, Biosynthesis pathway, Flavonoids

## Abstract

The Andean domesticated common beans (*Phaseolus vulgaris*) are significant sources of phenolic compounds associated with health benefits. However, the regulation of biosynthesis of these compounds during bean seed development remains unclear. To elucidate the gene expression patterns involved in the regulation of the flavonoid pathway, we conducted a transcriptome analysis of two contrasting Chilean varieties, Negro Argel (black bean) and Coscorron (white bean), at three developmental stages associated with seed color change, as well as different flavonoid compound accumulations. Our study reveals that phenolic compound synthesis initiates during seed filling, although it exhibits desynchronization between both varieties. We identified 10,153 Differentially Expressed Genes (DEGs) across all comparisons. The KEGG pathway ‘Flavonoid biosynthesis’ showed enrichment of induced DEGs in Negro Argel (PV172), consistent with the accumulation of delphinidin, petunidin, and malvidin hexosides in their seeds, while catechin glucoside, procyanidin and kaempferol derivatives were predominantly detected in Coscorrón (PV24). Furthermore, while the flavonoid pathway was active in both varieties, our results suggest that enzymes involved in the final steps, such as ANS and UGT, were crucial, inducing anthocyanin formation in Negro Argel. Additionally, during active anthocyanin biosynthesis, the accumulation of reserve proteins or those related to seed protection and germination was induced. These findings provide valuable insights and serve as a guide for plant breeding aimed at enhancing the health and nutritional properties of common beans.

**Supplementary Information:**

The online version contains supplementary material available at 10.1186/s12870-024-05593-5.

## Introduction

Legumes are one of the world’s most important sources of food supply. Among them, the common bean (*Phaseolus vulgaris*) is one of the most consumed. It is high in protein (rich in leucine, phenylalanine, and lysine), complex carbohydrates and good source of vitamins and minerals, while low in fat [1, 2]. Common bean is considered a functional food because it contains bioactive components such as phenolics, sterols and saponins [3–6]. The phenolic compound plays an important role in human health through its antioxidant activity, which is associated with anti-diabetic, anti-obesity, anti-inflammatory, anti-mutagenic and anti-carcinogenic properties [7]. In common beans, the phenolic compounds are present in the cotyledons and seed coat, being previously described phenolic acids, hydroxycinnamic acids and derivatives and flavonoids (flavonols, flavanones, flavanols, flavones and anthocyanins), among others [6, 8].

Flavonoids are synthetized by the general phenylpropanoids pathway and accumulated in the seed coat of common bean. A subclass of flavonoids are the anthocyanins, which are important in pigmented seeds. The most common anthocyanin found in colored common bean are the glycosides of delphinidin, petunidin, malvidin, and cyanidin [6, 9].

The biosynthetic pathway leading the anthocyanin accumulation is a highly conserved network and is an extension of flavonoid pathway [10]. The enzymes that catalyze each reaction step in anthocyanin biosynthesis are encoded by a group of structural genes divided into early and late [11]. The first group of genes are involved in the biosynthesis of downstream flavonoids. These include chalcone synthase (*CHS*), chalcone isomerase (*CHI*) and flavanone 3-hydroxylase (*F3H*). In certain species, the expression of these genes is never correlated with an increase in the anthocyanin concentration [12]. However, the late group of genes are required for the biosynthesis of specific flavonoids, including anthocyanin. This group includes flavonoid 3′-hydroxylase (*F3*′*H*), flavonoid 3′,5′-hydroxylase (*F3*′5′*H*), dihydroflavonol 4-reductase (*DFR*), anthocyanidin synthase (*ANS*), and UDP-glycosyltransferase (*UGT*). The dihydroflavonol biosynthesis step is considered a critical downstream branching point for the flavonoid metabolism. First, the formation of dihydroquercetin and dihydromyrcetin depends on F3’H and F3’5’H enzymes, respectively. These dihydroflavonols can be directed to the formation of anthocyanin or flavonols. Here, DFR compete with flavonol synthase (FLS) to catalyze the conversion of dihydroflavonols into flavonol or leucoanthocyanidin (anthocyanidin precursor), which are colored or uncolored compounds, respectively [13]. Further on, the anthocyanidin is glycosylated and methylated by UGT and O-methyl transferase (OMT), respectively [14, 15].

The expression of structural genes is differentially regulated by the interaction of R2R3 MYB transcription factors, basic helix-loop-helix (bHLH), and WD40 proteins. They interact in a complex known as MYB-bHLH-WD40 (MBW) [16, 17]. Other TFs have been reported that negatively regulate the anthocyanin biosynthesis, such as LvMYB1 in lily flowers and MtLAP1, which is responsible of anthocyanin biosynthesis or RH1 and RH2, that exert opposite effects binding to the MBW complex and determining anthocyanin leaf markings in *Medicago truncatula* [18, 19]. Other author reported that gene P, the master regulator of color in common bean encodes for a member of the bHLH proteins and belongs to the MBW complex [20, 21].

Some structural genes involved in the anthocyanin biosynthetic pathway have been described in kidney bean pods [22]. However, the transcriptional expression and regulation of the genes involved in this process have not been fully investigated in common bean seeds. With this aim, we performed a transcriptome comparison between two typical Chilean common bean landraces contrasting in the seed coat coloration, namely PV172 (Negro Argel) and PV24 (Coscorron Mendez). This research reports and correlates the flavonoid metabolites present in both varieties with regulated genes that leading to biosynthesis of anthocyanin in the black bean variety during the seed development and hypothesizes the role of regulatory proteins.

## Materials and methods

### Plant material

The common bean (*Phaseolus vulgaris* L.) varieties used in this study were provided by the Germplasm Bank Network of the Instituto de Investigaciones Agropecuarias (INIA). The varieties PV172 – Negro Argel (Acc N° QUI172) and PV24 – Coscorrón (Acc N° QUI24) were grown in a shelter facility at the INIA Quilamapu Research Center, Chillán (36◦34′ S; 72◦06′ O), Chile. Details of the facilities, establishment and growth conditions were previously described [23]. Seed samples were collected from plants grown under optimal irrigation conditions [23]. Approximately 300 flowers were tagged at anthesis. Pods were harvested every 3–6 days from anthesis [24]. Three phenological stages were defined per genotype, based on the color changes of the seeds. At the same time, per each phenological stage, seed samples were collected with three biological replicates. Collected samples were frozen with liquid nitrogen and stored at – 80 °C until total RNA extraction.

### Sample preparation for chemical measurement

Dry seeds of accessions PV24 and PV172 were processed separately [6]. Briefly, the dry seeds were powdered and extracted three times with 70% methanol containing 1% of formic acid in a 1:5 (w/v) ratio, under sonication for 20 min each time. After filtration, the extracts were combined and taken to dryness under reduced pressure. The crude extract was defatted three times with hexane in a 1:1 hexane: suspension ratio. After removal the remaining hexane, the aqueous phase was mixed with activated Amberlite XAD-7 resin and stirred for 30 min. After filtration and washing, the compounds adsorbed in the resin were desorbed with MeOH containing 1% formic acid. Then, the solution was dried to afford the enriched extract, which was used for analyses.

### HPLC-MS/MS analysis

The main anthocyanins in the enriched extracts were separated and tentatively identified by UHPLC/HPLC-DAD using a Bruker Elute LC system coupled to Q-TOF Compact detector (Bruker Daltonics, Bremen, Germany). The column used was a Kinetex PFP 100 Å, LC column (250 mm × 4.6 mm, 5 μm particle size, Phenomenex, Torrance, CA, USA). The gradient solvent system consisted of 0.1% formic acid in water (solution A) and 0.1% formic acid in methanol (solution B) as follows: 0–3 min, 2% B; 3–33 min, 2–80% B; 33–35 min, 100% B; 35–39 min, 100% B. The flow rate was 0.3 mL/min. The injection volume was 20 µL, and the column temperature was 30 °C. Samples were monitored at 520 nm. Data was acquired in the full scan mode (range of *m/z* 200–2000) in the positive ionization mode. The MS^n^ mode was used in the range of 100–1000 *m/z* (scan 0.2 s centroid mode). Sodium formate (10% formic acid, 1 M) was used as internal calibration with a mass accuracy < 3 ppm. The compounds were identified by comparison of the molecular formula, fragmentation pattern, visible and UV spectra, and literature, including the database from the Metabolomics Innovation Center (https://foodb.ca/).

### Seed RNA purification and RNAseq analysis

To obtain purified RNA from common bean seed, the starch in the seeds must to be removed. This procedure is necessary to prevent solidification of samples in the guanidine isothiocyanate (GITC)-based RNA extraction kit.

The RNA extraction was carried out from 100 mg of seed, which was grounded in a mortar with liquid nitrogen. The flour sample was transferred to a prechilled 1.5 mL RNAse-free microcentrifuge tube. An aliquot of 450μL of extraction buffer (100mM TRIS pH = 8.0, 150mM LiCl, 50mM EDTA, 1.5% sodium dodecyl sulfate (SDS), 1.5% 2-mercaptoethanol) was added to the flour sample and mixed vigorously in a vortex [25]. Then, an aliquot of 450μL of phenol-chloroform (1:1, pH = 4.7) was added and mixed by inversion. The tube was centrifuged at 13,000 g for 15 min at 4 °C. The upper aqueous phase was transferred to a new tube and carefully mixed with 400μL of RNA Lysis Buffer from Quick-RNA Mini prep (Zymo Research, CA, USA). The protocol was continued as described in the RNA Kit. The RNA purity was evaluated using Nanodrop TM 1000 Spectrophotometer (Thermo Scientific) and the RNA integrity was evaluated using a 2100 Bioanalyzer instrument (Agilent, 5301 Stevens Creek Blvd. Santa Clara, CA 95051, USA), accepting values > 6.3 with smooth baseline.

The sequencing service was provided by Novogene Corporation Inc. (2921 Stockton Blvd., Suite 1810, Sacramento CA 95817; USA). Directional mRNA library preparation (poly A enrichment) was performed. Samples were sequenced using the platform NovaSeq 6000 (Illumina Inc., 5200 Illumina Way, San Diego, CA 92122, USA) for 150 bp fragment in pair-end.

### Quality control, mapping and transcriptome assembly

Raw sequencing data were subjected to quality control and trimming to remove adapters and low quality reads. For this, the FastQC application v0.11.9 [26], was used to determine the raw read quality of each sequenced library. Then, the low quality reads (quality mean less than Q30), reads less than 50 pb in length, and reads containing adapters were then removed using the Trimgalore tool v.0.6.7 (https://www.bioinformatics.babraham.ac.uk/projects/trim_galore/). All downstream analyses were based on the high-quality data (clean reads) obtained from the previous steps. Clean reads were alignment to *Phaseolus vulgaris* genome using HISAT2 (v2.2.1) [27], while the mapped reads were assembled using StringTie (v2.1.1) [28]. Both tools were used with default parameters. The *Phaseolus vulgaris* genome used in this study corresponded to the assembled version 2.0, with the annotated version: 2.1, downloaded from www.phytozome.net.

### Differentially expressed genes (DEGs)and enrichment analysis

The count reads to estimate the gene abundance of each library was performed using StringTie, while the differentially expressed genes between libraries were estimated using the edgeR package of the R software [29]. The counts were normalized mediating the calculation of FPKM, using the cufflinks software version 2.2.1. The differentially expressed genes (DEGs) were obtained considering a false discovery rate (FDR) parameters below 0.05 and absolute fold change ≥ 2.

The list of genes selected from each group was analyzed for GO terms and KEGG pathway enrichment of DGEs. The DEGs were grouped according to of the ontology categories: molecular function and biological process or KEGG pathway using Legume IP V3 [30]. The p-values were < 0.05 and corrected by *Benjamini–Hochberg* adjustment for multiple hypothesis testing.

### Proteins and genes related to metabolites pathways

The metabolites identified were correlated with the related protein available from Uniprot using Proteome of *A. thaliana* (taxid: 3702) as reference organism. The local alignment to *P. vulgaris* genome v2.1 was performed using Diamond [31] maintain only the best result for each gene from *P. vulgaris*.

### DEGs progression related to metabolites pathways

From the DEGs, filter the samples for PV172 and PV24 and compared the same steady C1, C2, and C3 was performed. These DEGs were searched in the metabolite pathway associated to *A. thaliana* putative ortholog. Then, the Log_2_FC were associated with each ortholog and the sum of the values were separate in the positive FC (increase), and the negative FC (decrease). It was plotted together with altair library [32]. The processes to generate tables and plots were programmed using pandas [33] and Python language.

### Realtime qRT-PCR analysis

The cDNA synthesis was performed from 2 µg of total RNA for each sample using oligo (dT) using GoScript™ Reverse Transcriptase (Promega Corp., 2800 Woods Hollow Road Madison, WI 53711 USA) according to the manufacturer’s instructions. Gene transcript levels of candidate genes were measured by quantitative PCR (RT-qPCR) using an AriaMx Real-Time PCR System (Agilent, 5301 Stevens Creek Blvd. Santa Clara, CA 95051, USA). Reactions were performed in triplicate (technical replicates) using the 5x HOT FIREPol EvaGreen qPCR Mix Plus (Solis Biodyne, Teaduspargi 9, 50411 Tartu, Estonia) according to the manufacturer. The relative expression patterns of the target genes were calculated using the 2 − ΔΔCT method repeated in triplicate [34], and normalized against PvACT11 (Phvul.008G011000) gene expression. Primers used for qPCR analysis are listed in the Table S1.

## Results

### Seed develops and color change

**Fig. 1:**
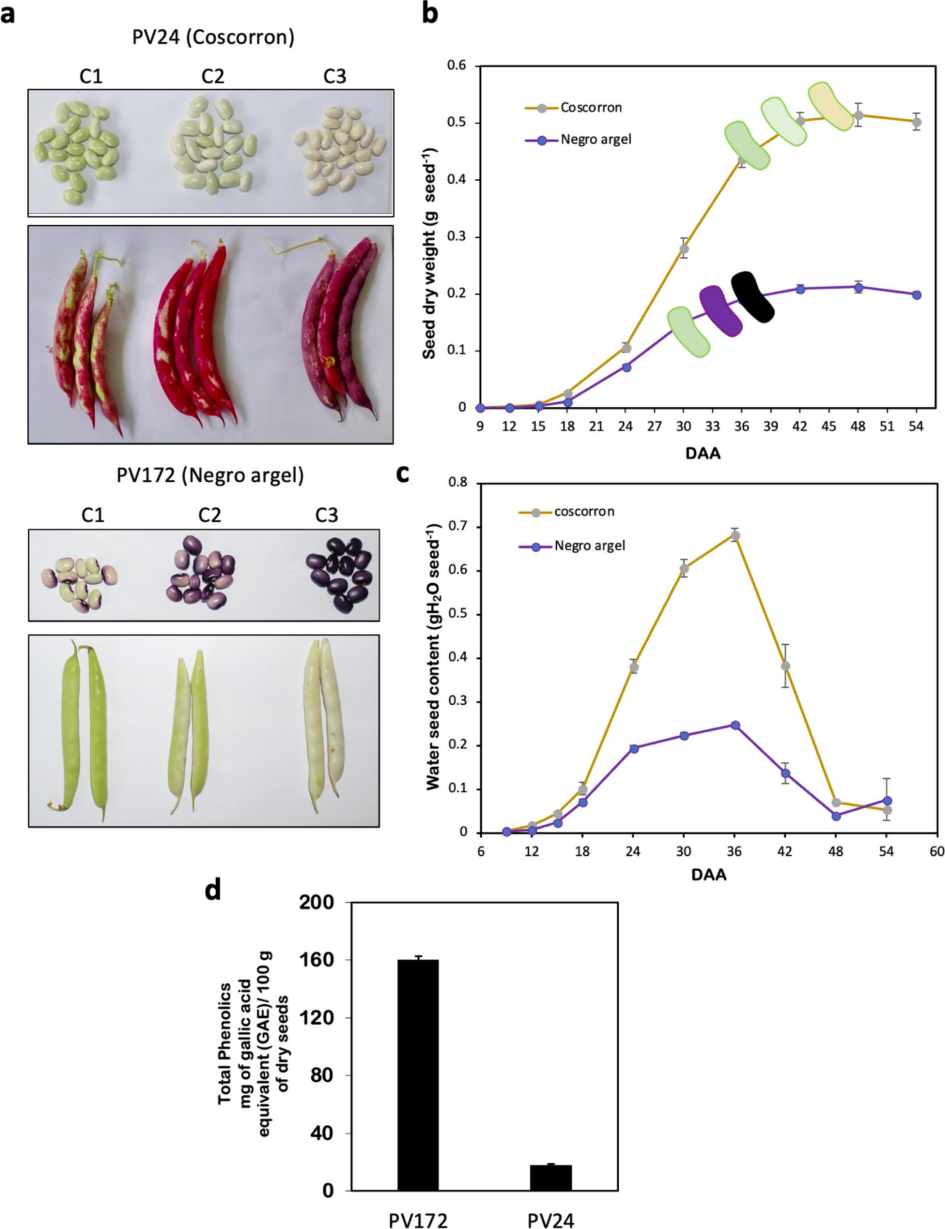
Seed development and color acquisition in *Phaseolus vulgaris* varieties PV24 and PV172. Measurement of (**a**) seed dry weight (SDW) and (**b**) water seed content from nine days after anthesis. Mean ± SEM, *n* = 21. (**c**) Stage involved with color change in PV24 and PV172 named as C1, C2 and C3. (**d**) Total phenolics compounds for PV172 and PV24 varieties from complete seed

To identify the genes responsible for anthocyanin synthesis in the black bean varieties the three phenological stages selected in contrasting varieties started with the green color and finished with the black or white seeds, for PV172 and PV24, respectively (Fig. 1a). The measurement of seed development from anthesis was evaluated by the dry weight and water accumulation in the seed for the 54 days from anthesis (Fig. 1b, c). The measurement started nine days after anthesis (DAA), showing exponential growth in dry weight from 18 DAA for both varieties until 30 DAA in PV172 or 36 DAA for PV24 (Fig. 1b). The exponential increase of dry weight coincided with the increase in water content which was stopped from 36 DAA (Fig. 1c). Both varieties reach the maximal dry weight accumulation from 39 DAA, when the water seed content start to decrease (Fig. 1c). The change of color start at 24 DAA for PV172 but at 30 DAA for PV24. The change of color in PV172 started from hilum weakly purple increasing until black coloration at day 30 (Fig. 1a, b). The accumulation of phenolics compound, mainly composed by anthocyanins was 9 times higher in PV172 compared with PV24 (Fig. 1d). In PV24, the change of color from green to white occurs until the 45 DAA. The change of seed color is coincident with the pod ripening. The pods turn from green to whitening in PV172 and completely red in PV24 (Fig. 1a). The pod in the third phase starts a dehydration process.

### Identification and quantification of phenolic compounds

Quantification of various phenolic compounds was performed. Ferulic acid, which is part of the phenyl-propanoid pathway, was identified only in PV24 (Table 1). Regarding to the elements of the flavonoid pathway flavonols such as kaempferol 3-O-glucoside and kaempferol hexoside pentoside were the most abundant detected in both varieties, while kaempferol and kaempferol acetyl glucoside were only detected in PV24. Quercetin hexoside pentoside was found only in PV172, but was the most abundant flavonol with 7.16 mg/g DW. Flavan-3-ols such as catechin glucoside and procyanidins were only identified only in PV24. At the same time, six anthocyanins were identified in the extracts from PV172 but they were not detected in PV24. Previously, we reported that PV172 included three malvidin, two delphinidin and one petunidin derivatives (Table 1, adapted from Nina et al. [6]). Three malvidin glycosides were characterized by the [M + H] + signal at m/z 331 amu, including two hexosides and a dihexoside. Delphinidin-3-hexoside was identified by the [M + H] + signal at m/z 465 and the neutral loss of 162 amu leading to the aglycone at m/z 303 amu. The Delphinidin-3-hexoside was the most abundant anthocyanin with a concentration of 47.66 mg/g dry weight. Petunidin hexoside was identified by the [M + H] + signal at m/z 479 and the MS2 signal at m/z 317 amu. It was the second most abundant anthocyanin in PV172 reaching a concentration of 26.15 mg/g dry weight. A compound presented a neutral loss of 248 amu, an additional 86 amu to the neutral loss of the glycoside (162 amu), indicating the presence of a malonyl group as a substituent. The compound was tentatively identified as delphinidin malonylhexoside (Table 1, Table S4).

**Table 1:** Quantification of main phenolics in PV172 and PV24 common bean varieties, expressed as mg/100 g of dry seeds

Compound	PV24	PV172
**Hydroxycinnamic acids**		
Ferulic acid	0.18 ± 0.01	ND
**Flavonols**		
Kaempferol 3-*O*-glucoside	3.37 ± 0.03	2.64 ± 0.03
Kaempferol hexoside pentoside	1.83 ± 0.02	2.71 ± 0.04
Kaempferol acetyl glucoside	1.07 ± 0.01	ND
Kaempferol	0.11 ± 0.01	ND
Quercetin hexoside pentoside	ND	7.16 ± 0.09
**Procyanidins**		
Catechin glucoside	9.70 ± 0.03	ND
Procyanidin trimer B	3.95 ± 0.05	ND
Procyanidin dimer B	0.44 ± 0.02	ND
**Anthocyanins**		
Malvidin dihexoside	ND	1.93 ± 0.05
Delphinidin 3-hexoside	ND	47.66 ± 1.62
Petunidin 3-hexoside	ND	26.15 ± 0.99
Malvidin 3-hexoside 1	ND	0.95 ± 0.02
Malvidin 3-hexoside 2	ND	13.70 ± 0.71
Delphinidin-3-(6’’-malonylhexoside)	ND	4.65 ± 0.17
Total anthocyanins	-	95.04

### Transcriptome sequencing and analysis

From the RNAseq analysis, 819,672,804 raw reads were obtained among the 18 libraries, with an average of 45,537,378 raw reads and GC content of 46.9% per library (Table S2). After the pre-processing step, 784,841,100 (95.7%) clean reads passed the quality control (no artefacts, length > 50 pb, Quality = 30), with an average of 43,602,283 (95.7%) clean reads per library. The 18 libraries were mapped to the *Phaseolus vulgaris* genome with an average of 98.93% of mapped reads (Table S2).

**Fig. 2:**
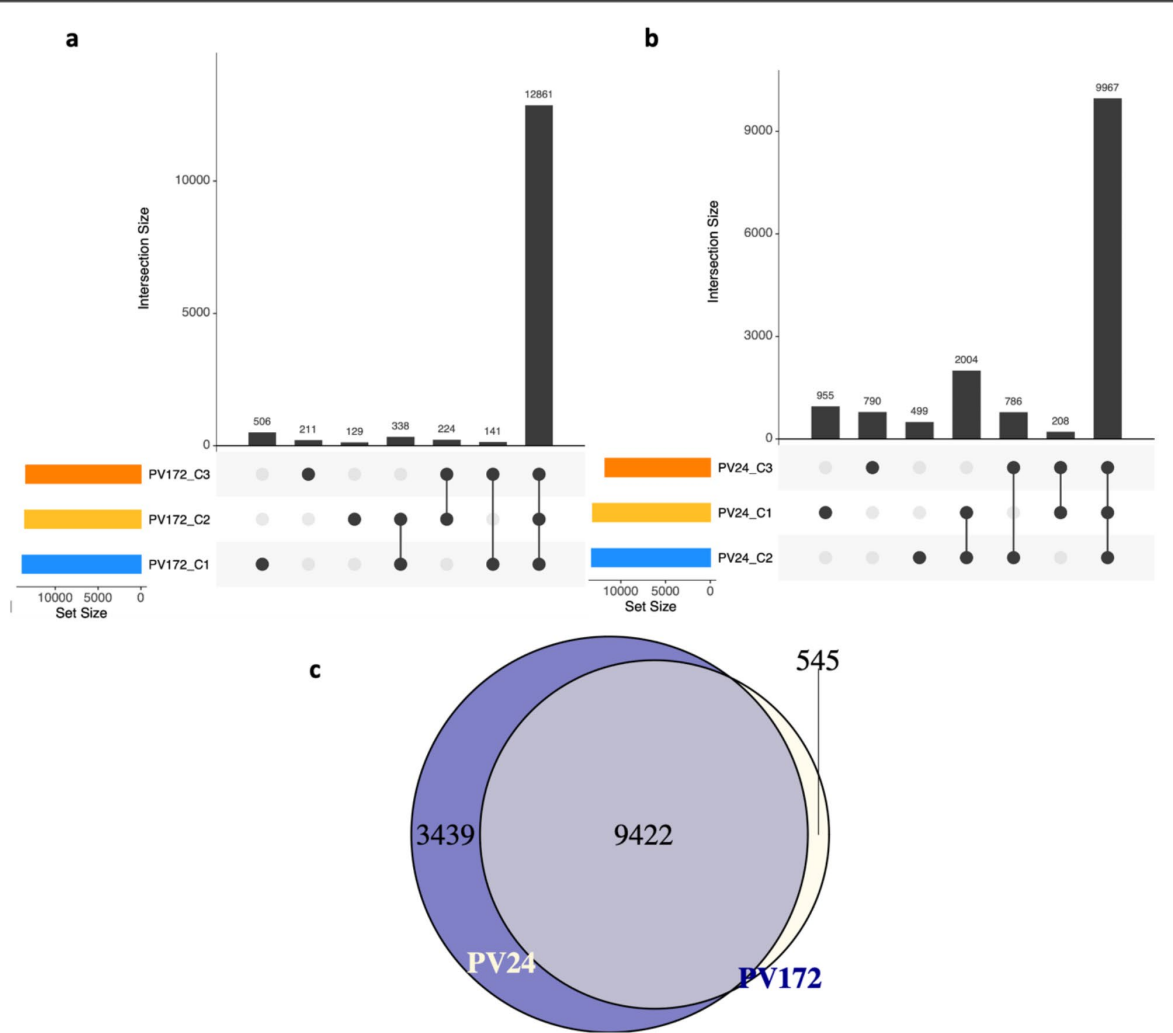
Venn diagram for expressed genes (**a**) PV172 and (**b**) PV24. Treatment consisted from C1, C2 and C3 that correspond to the three stages of seed development. (**c**) Venn diagram for shared genes in the three stages of PV172 and PV24

Based on the expressed genes (FPKM > 1), the 89.3% (12,861 genes) of genes in PV172 were shared among the three developmental stages (Fig. 2a), whereas 65.5% (9,967 genes) were shared in PV24 (Fig. 2b). Between a 0.9–6.3% of genes were expressed exclusively in one stage. In PV172 more genes were exclusively shared between PV172-C1 and PV172-C2 than PV172-C2 and PV172-C3 or PV172-C3 and PV172-C1 (Fig. 2a). Similar results were observed for PV24 (Fig. 2b). A total of 12,861 and 9,967 genes were identified among the three biological replicates for PV172 and PV24, respectively; 9,422 genes were shared among the two varieties in the three developmental stages (Fig. 2c). Additionally, 70.3% (9,422 genes) were common in both varieties. Interestingly, 25,7% of genes were exclusively expressed in PV172 while 4.1% were only found in PV24 (Fig. 2c).

### Differentially expressed genes

**Fig. 3:**
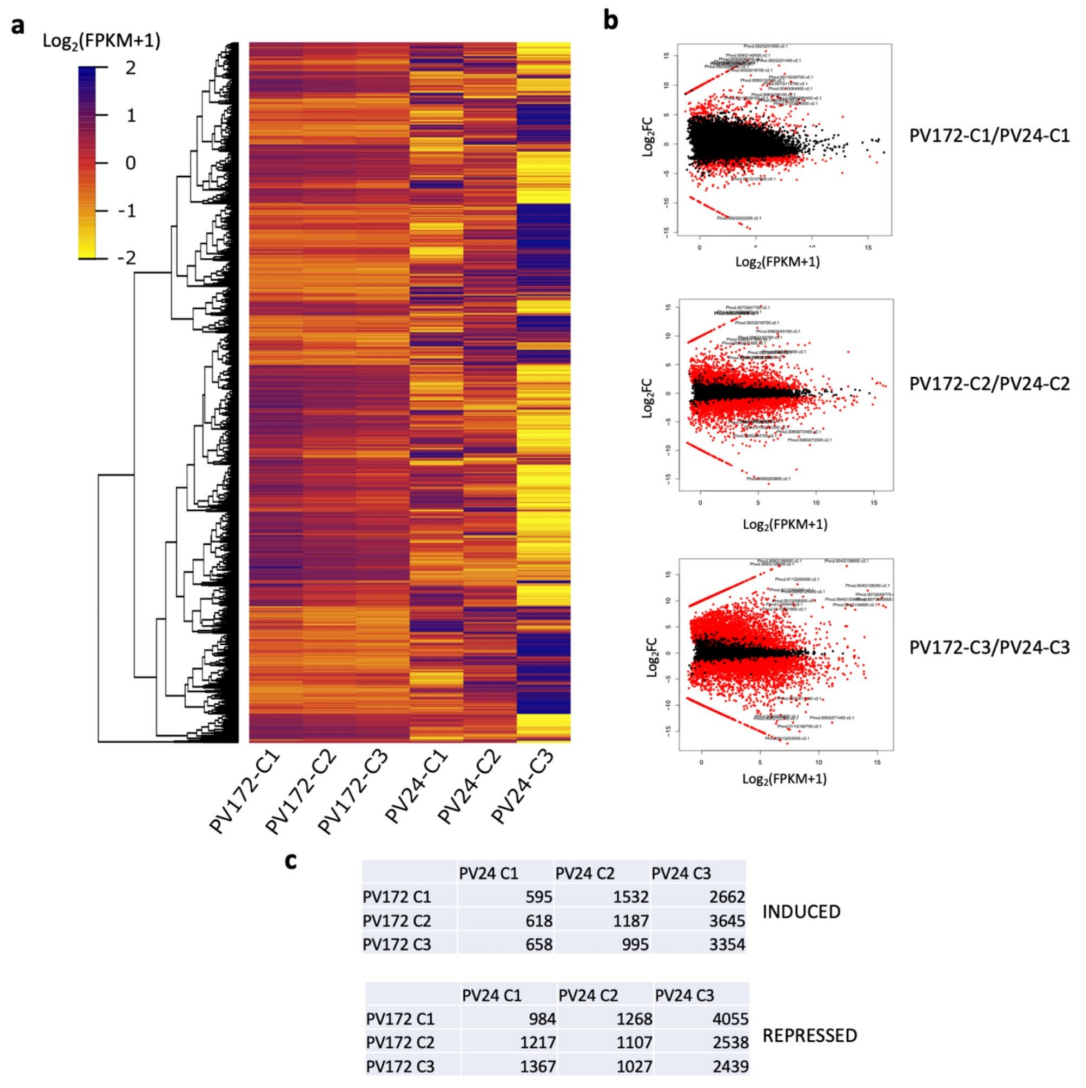
Heatmap of differentially expressed genes for all the comparison between PV172 and PV24. (**a**)10,153 DEG were obtained from all the comparison with a criteria of FDR < 0.05 and − 2 < Log(FC) > 2. The values correspond to Log_2_(FPKM + 1). (**b**) Volcano plots representation for specifical comparisons C1, C2 or C3 stages, using a fold change cutoff = 2. (**c**) Table of induced and repressed genes for comparisons between PV172/PV24

Significant differentially expressed genes (DEGs) were tested in both varieties PV172 and PV24 between the three seed stages C1, C2 and C3. Here, a number of 10,153 DEGs were found considering all the comparisons, using the criteria of FDR < 0.05 and − 2 < Log(FC) > 2 (Fig. 3a).

The comparison between the varieties was performed for specific developmental stages. The number of induced DEGs was lower in PV172-C1 compared with PV24-C1 (Fig. 3b and c), but the DEGs were increasing for the stages C2 and C3 (Fig. 3b and c). The higher number of DEGs was found between PV172 stages and third stage of PV24 (PV24-C3). Similar trends were observed for repressed genes, where PV172 showed the higher number of genes compared with PV24-C3 (Fig. 3c).

### GO and KEGG pathway enrichment analysis

**Fig. 4:**
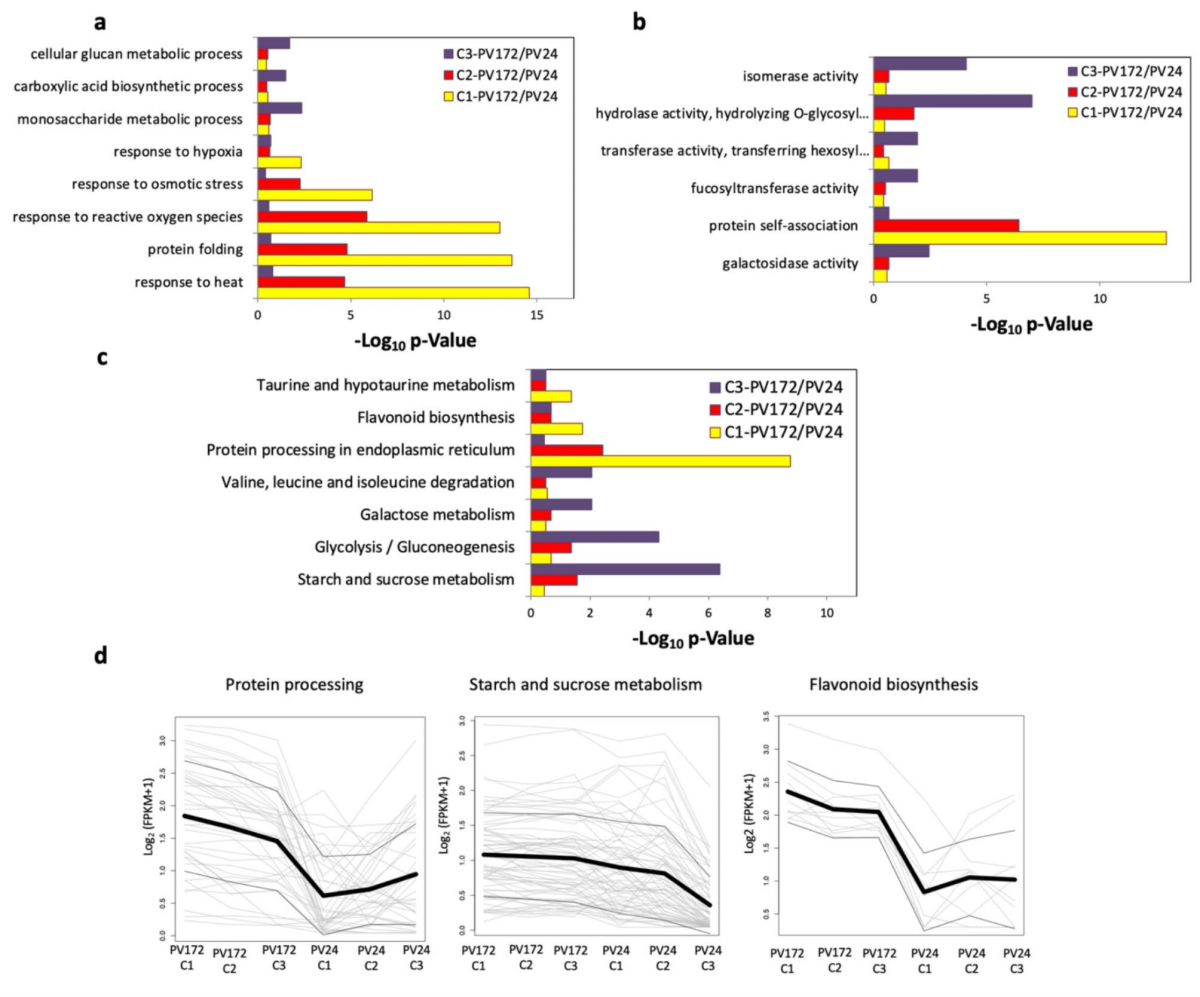
Gene ontology and KEGG pathway functional classification. The DEG between PV172/PV24 from equivalent stages were filtered from Log(FC) > 2 and FDR < 0.05. The GO categories considered were (**a**) Biological process, (**b**) Molecular function, including also the (**c**) KEGG pathways. Selected GO and KEGG pathways were selected considering a p-value < 0.05 and non-redundant relationship according enrichment analysis. (**d**) Gene expression for groups of KEGG pathways: protein processing, starch and sucrose metabolism and flavonoid biosynthesis

To investigate the biological function of the DEGs induced in PV172/PV24, GO and KEGG pathway enrichment analyses were performed. The biological processes (BP) “response to heat”, “response to reactive oxygen species”, “response to osmotic stress”, and “response to hypoxia” were enriched in C1 and C2 stages, while “cellular glucan metabolic process”, “carboxylic acid biosynthetic process”, and “monosaccharide metabolic process” were weakly enriched in C3 stage (Fig. 4a). The BP “protein folding” was related to with “protein self-association” from molecular function (MF) which were mainly consisted of genes encoding for heat shock proteins and was highly enriched in C1 stage (Fig. 4a, b). GO categories from MF such as “galactosidase activity”, “fucosyltransferase activity”, “transferase activity, transferring hexosyl groups” and “hydrolase activity, hydrolyzing O-glycosyl compounds” were enriched in C3 stage in a similar way than the KEGG pathways “Galactose metabolism”, “Glycolisis/Gluconeogenesis”, and “Starch and sucrose metabolism” (Fig. 4b, c). Additional KEGG pathway “Protein processing in endoplasmic reticulum” was enriched in C1 and C2 stages and maintain a high expression in PV172 compared with PV24 (Fig. 4d). The categories “flavonoid biosynthesis” and “taurine and hypotaurine metabolism” were enriched only in C1 stage. However, the expression of enriched genes for “flavonoid metabolism” were higher in PV172 stages than PV24 (Fig. 4c).

The KEGG pathway “Starch and sucrose metabolism”, “Galactose metabolism”, “Valine, leucine and isoleucine degradation” and Glycolysis/Gluconeogenesis were enriched only in C3 stage. The expression of genes for KEGG pathway “Starch and sucrose metabolism” maintained a similar Log_2_(FPKM + 1) between the stages in PV172, but decreased in PV24 C3 (Fig. 4c).

The expression of genes involved in KEGG pathways “Protein processing” was mainly induced in PV172 stages. The expression of genes for “Starch and sucrose metabolism” was high in PV172, decaying more severely in PV24 C3. The expression of “flavonoid biosynthesis” genes was induced in PV172 respect to PV24(Fig. 4d).

**Fig. 5:**
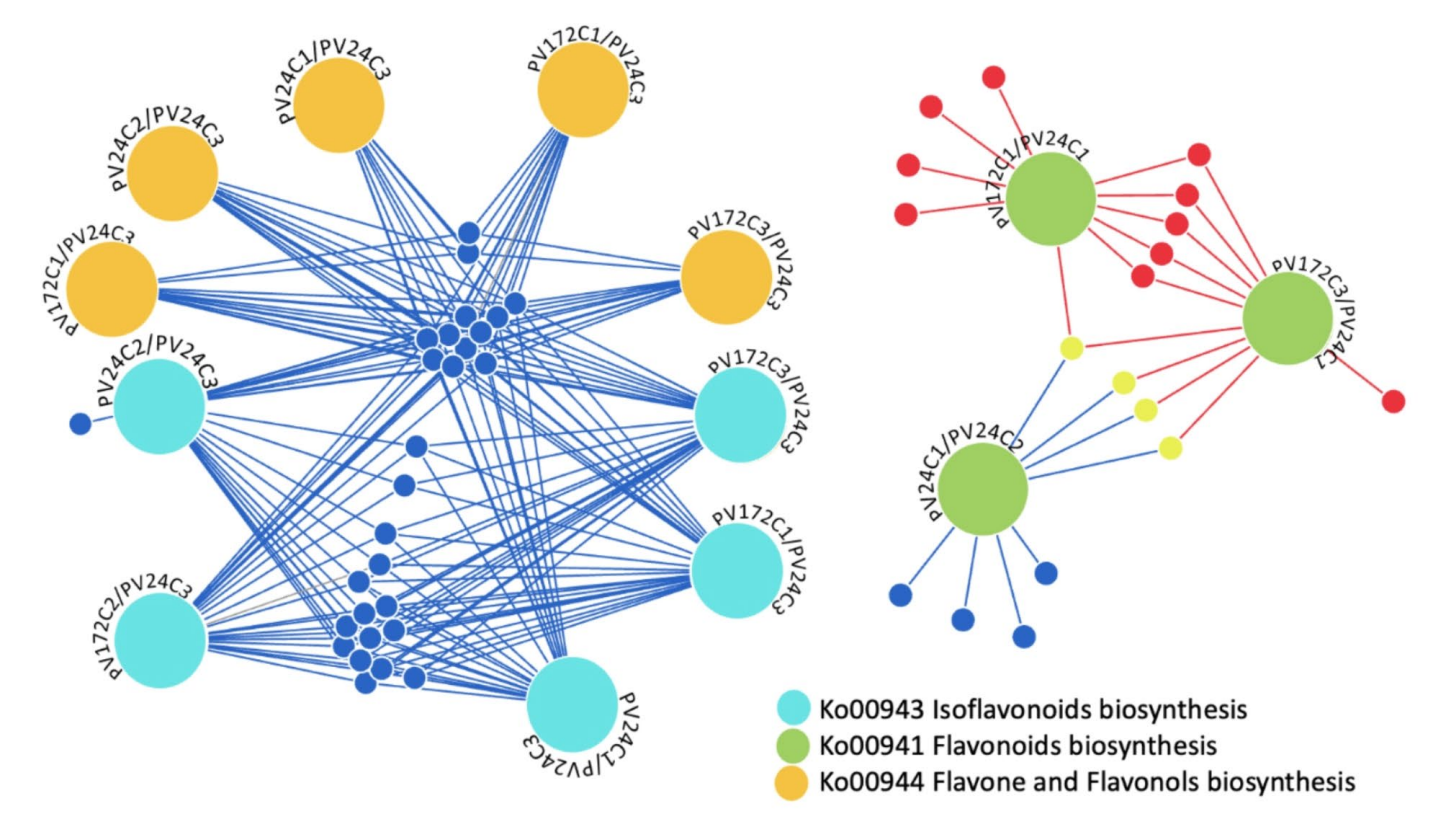
Flavonoid, Isoflavonoid and flavones enrichment in stages of PV172 and PV24 varieties. DiVenn subset analysis of genes from the KEGG pathways: (Ko00943) Isoflavonoids biosynthesis, (Ko00941) Flavonoids biosynthesis and (Ko00944) Flavone and Flavonols biosynthesis. Red circle: induced genes, yellow circle: induced in one condition and repressed in the other condition; blue circle: repressed genes

KEGG pathway analysis related to flavonoids was performed for all the possible comparisons between PV172, PV24 and the three developmental stages. The KEGG pathway “Flavonoid biosynthesis” was enriched for induced DEGs in PV172-C1 or C3 over PV24-C1, while some of the same genes were repressed during PV24-C1/PV24-C2 (Fig. 5). Three of them were encoded to chalcone synthase. The KEGG Pathway “Isoflavonoid biosynthesis” and “Flavonone and Flavonols biosynthesis” were enriched in the repressed group of DEGs. These genes were repressed in all the PV172 stages compared to PV24-C3, but additionally some of them were repressed in PV24 in favor of earlier stages (Fig. 5). Among them, eight genes encode for isoflavone-7-O-beta-glucoside-6’’-O-malonyltransferase (2.3.1.115). Also, genes related with isoflavonoid biosynthesis included Isoflavonoid synthase (1.14.13.136) and Isoflavone 2’-monooxygenase (1.14.13.89) (Table S3).

### Flavonoid biosynthesis genes

**Fig. 6:**
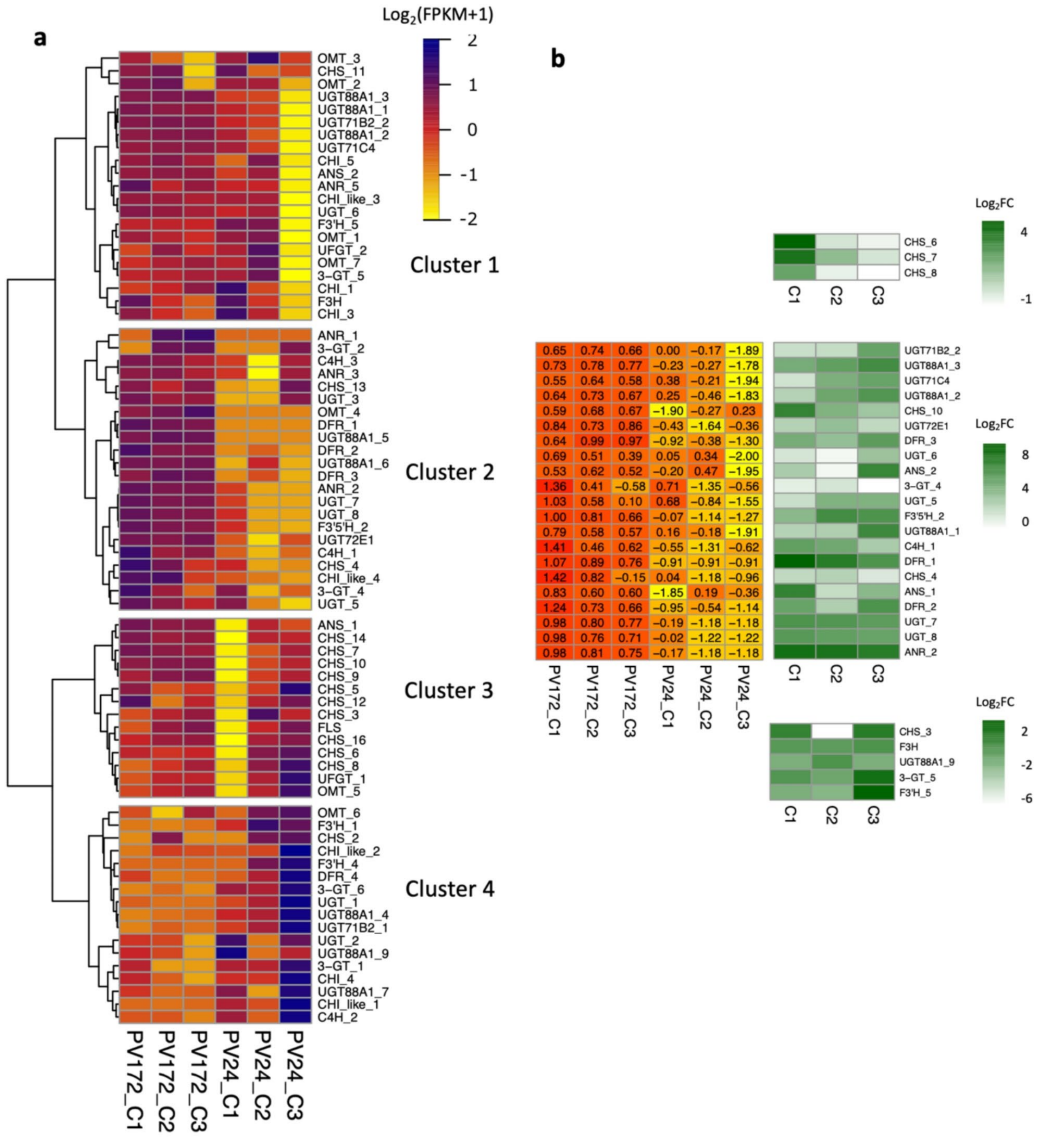
Heatmap for flavonoid biosynthesis genes in PV172 and PV24 varieties of *Phaseolus vulgaris*. (**a**) Heatmap for comparison between PV172 and PV24. The expression values were standarized as Log_2_(FPKM + 1), centered and scaled in the row direction and clustered using warD2. Specific cluster that include DEGs (LogFC > 2, FDR < 0.05) and related with anthocyanin biosynthesis was detailed in (**b**) Here correspond to develop stages C1, C2 and C3 comparison between PV172 and PV24

The annotation of anthocyanin-related genes was performed identifying 87 genes in the *Phaseolus vulgaris* genome (Table S3). Among them, 74 genes were present at least in one of the stages with higher expression, forming four major clusters (Fig. 6a). The clusters 3 and 4 included 31 genes that were mainly present in PV24 C2 or C3. These two clusters include eleven genes coding for naringenin-chalcone synthase (CHS; EC:2.3.1.74), but also some genes coding for chalcone isomerase (CHI; EC:5.5.1.6) and UDP-glycosyl transferase (UGT, EC 2.4.1.-), among others related with flavonoid biosynthesis. The first cluster grouped 21 genes expressed in PV24-C1 and C2 but also in PV172 stages. A gene encoding for F3H, which is also important in anthocyanin biosynthesis, was clustered here (Fig. 6a, b).

Twenty-two genes were highly expressed in PV172 stages respect to PV24 and were grouped in the second cluster. Several of them were also significantly induced in PV172 (Fig. 6a). Totally, among flavonoid biosynthesis genes expressed in these two varieties, twenty-one genes were significantly induced in PV172 (Log2(FC) > 2, FDR < 0.05) compared to the corresponding stage in PV24 (Fig. 6b). In particular, the genes *CHS_4* and *CHS_10* (Phvul.001G083000 and Phvul.002G039000) were induced in PV172 compared with PV24. Eight genes encoding for chalcone isomerase (CHI, EC:5.5.1.6) or CHI-like proteins were identified. Some CHI genes are present in all stages in both varieties, but none of them was specifically induced in PV172 compared to PV24 (Fig. 6b). One gene encodes for naringenin dioxygenase (F3H, EC 1.14.11.9) which is highly expressed in both varieties (Phvul.003G261900) but not induced in one stage specifically. Here, we annotated two genes encoding for flavonoid 3’,5’-hydroxylase (F3’,5’H, EC:1.14.14.81), but only one of them (F3’,5’H_2, Phvul.006G018800) was induced in PV172 (Fig. 6b). On the other hand, five genes were annotated coding for Dihydroflavonol 4-reductase (DFR, EC:1.1.1.219) (Table S3), where 3 are overexpressed in PV172 respect to PV24 during the three development stages (*DFR_1*, Phvul.001G012700; *DFR_2*, Phvul.001G012800; *DFR_3*, Phvul.005G090200). The next enzyme involved in the anthocyanin biosynthesis is anthocyanidin synthase (ANS, EC 1.14.20.4). Two genes encoding for ANS which were induced in PV172 (*ANS_1*, Phvul.002G152700, *ANS_2*, Phvul.010G000300) (Fig. 6b).

In addition, 32 encoding genes to UDP-glycosyltransferase (EC 2.4.1) were annotated in *Phaseolus vulgaris* genome according to the Phytozome database (Table S3). Twenty-six of them are present in the common bean seed for some of the stages with a probable role in flavonoid glycosylation (Fig. 6A). Eleven of them were differentially induced in PV172 (*UGT71B2_2*: Phvul.010G053700; *UGT88A1_3*: Phvul.004G138450; *UGT71C4*: Phvul.008G262000; *UGT88A1_2*: Phvul.004G138400; UGT*72E1*: Phvul.002G026900; *UGT88A1_1*: Phvul.004G137900; *UGT_6*: Phvul.011G136700; *UGT_5*: Phvul.011G136400; *3GT_4*: Phvul.004G104200; *UGT_7*: Phvul.005G093500; *UGT_8*: Phvul.005G093600). Also, one of three cinnamate 4-hydroxylase (C4H, EC 1.14.14.91) was induced in the black bean (*C4H_1*, Phvul.007G026000). Finally, eleven genes encoding for o-methyltransferase (OMT; EC 2.1.1.104) were identified, being present in some of the stages in PV24 or PV172 (Fig. 6a, Table S3). Among them, *OMT-7* is the only that maintains a high expression in PV172 as well as in PV24, although without a significantly induction between both varieties. Other genes were also related with flavonoids biosynthesis such as Anthocyanidin reductase (ANR; EC:1.3.1.112), Coumaroyl-CoA: anthocyanidin-3-O-glucoside-6’’-O-coumaroyltransferase (AT; EC:2.3.1.-) and Malonyl-CoA: anthocyanidin-5-O-glucoside-6’’-O-malonyltransferase (5MAT; EC 2.3.1.-) (Fig S4 and Table S3).

A high correlation was found among anthocyanins accumulation in PV172 and expression of DEGs related with this pathway, mainly during the C1 stage, coincidently with flavonoids accumulation. The accumulation of flavonoids, kaempferol and kaempferol 3-glucoside was correlated in PV24 with DEGs from C2 and C3 stages. The quercetin derivative accumulation was correlated with DEGs induced in PV172 from C1 to C3 stages (Fig S4 and Table S5).

**Fig. 7:**
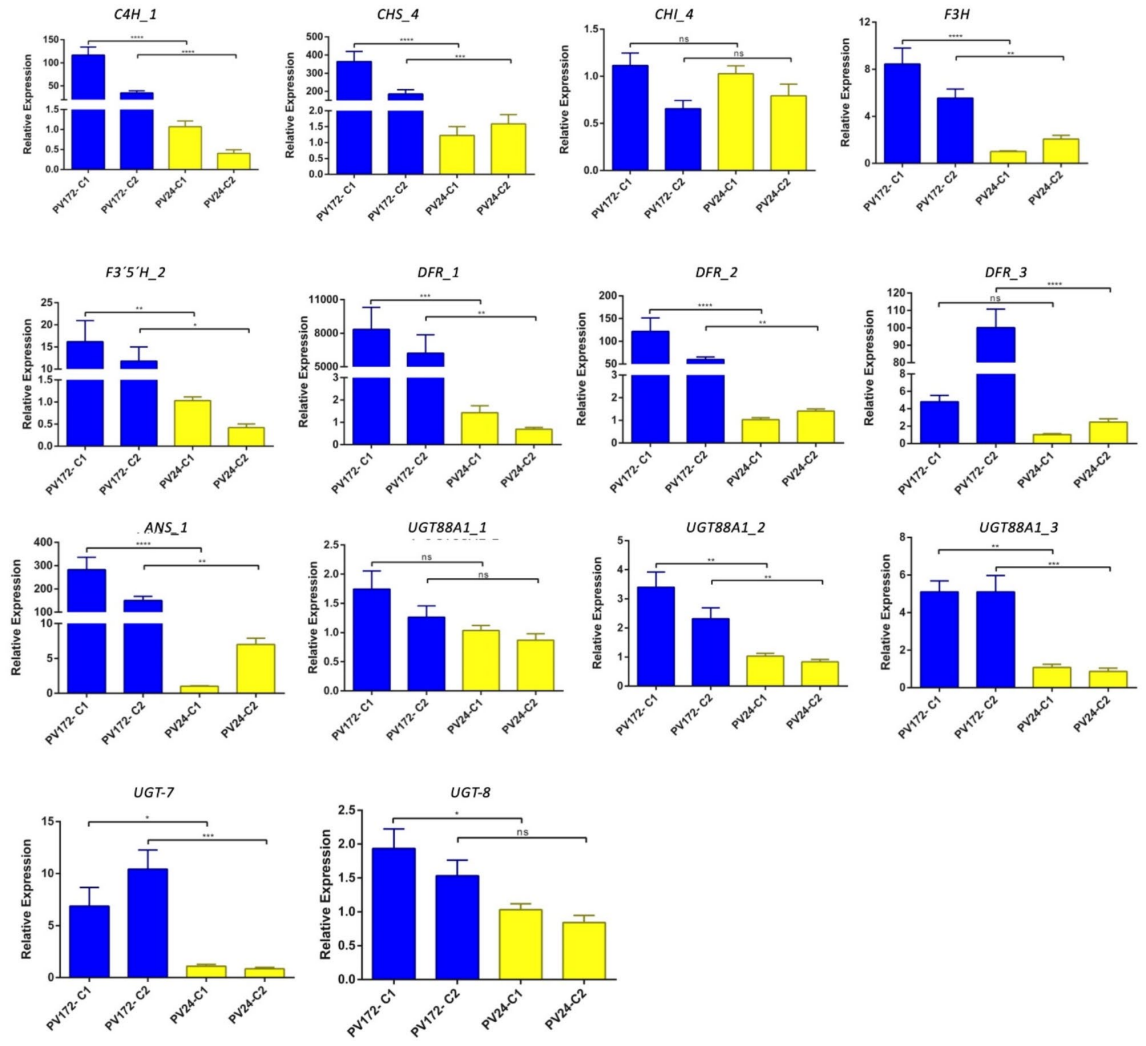
Relative gene expression of flavonoid biosynthesis genes in the skin of *Phaseolus vulgaris* PV172 and PV24 Chilean varieties at two distinct phenological stages. Relative transcript levels are shown as ΔΔCt values. Data show means and standard error of the mean (SEM) of three biological replicates. Asterisks indicate a significant difference between PV172 and PV24 at the same phenological stage using one-way analysis of variance, Tukey post-hoc test ***P < 0.0001

In order to validate the RNAseq results, some genes related to anthocyanin biosynthesis were analyzed by qRT-PCR. Similar trends were observed for the evaluated genes (Fig S1). The genes were analyzed under two of the development stages and showed in the seed coat. The expression respect to the complete seed was considerably increased for some of the genes (Fig. 7). The genes *C4H_1*, *CHS_4*, *DFR_1*, *DFR_2*, DFR_3 and *ANS_1* were induced by fifty folds for seed coat in PV172 with respect to PV24, a higher value compared with to the whole seed. However, the gene encoding for CHI_4 did not show significant changes between both varieties. The gene *F3’5´H_2* was highly expressed in complete seed at C1, C2 and C3 in PV172, compared with PV24 and these relative values were maintained when evaluated in seed coat. The expression of *UGT88A1_2* and *UGT88A1_3* was significantly higher for PV172 in seed coat. However, a higher induction was found for *UGT88A1_2* in the whole seed. The expression of *UGT-7* was also significantly higher in PV172, whereas in the expression of *UGT-8* was not clear (Fig. 7).

### Regulatory genes

We identified 23 genes encoding for TFs or protein domains with transcriptional activator function, which were significantly and differentially expressed between both varieties. Also, we explored the most important orthologous genes involved in regulation of anthocyanin biosynthesis. The orthologous for TT8 in *P. vulgaris* was Phvul.007G171333, but it is not induced in PV172 under the stages evaluated. The TF *MYB113* (Phvul.008G038400) was not expressed in the PV172 and PV24 transcriptomes, while orthologous *WD40* domain protein (Phvul.009G129300, Phvul.004G026700) were not induced in PV172. Among the identified regulatory genes three clusters were found (Fig S2). Two of them were highly overexpressed in PV172 seed stages. A homolog to SPATULA TF and PIF, an agamous-like MADS-box protein (Phvul.003G189100), a Myb/SANT-like DNA-binding domain (Phvul.007G224600), and a MBF1 multiprotein-bridging factor 1 (Phvul.004G162100) were induced in PV172. Other genes encoding TFs such as PLATZ, Brevis radix-TF, GATA, TIFY 10 A MYB26 and bZIP TF were also induced in PV172 (Fig S2).

## Discussion

Three main phases characterize seed development in legumes. A pre-maturation phase is associated with high mitotic division, while the second phase is focused on the with reserve accumulation and seed weight gain. This second phase is also known as the onset of seed filling. The third phase is the desiccation, characterized by a water loss and accumulation of seed protection proteins. Therefore, while the initial phase lasts up to 15–18 DAA and is associated with a low gain in DW and moisture, but with a high cell division, it continues with a rapid growth phase that lasts until 36–42 DAA and the final constant DW phase thereafter [24]. These phases are evidenced in the kinetics of seed dry weight in the two varieties of Chilean common bean but also in the water content along of the seed development (Fig. 1).

The aim of this research was analyzing the flavonoid composition present in the contrasting coloring varieties PV172 (Negro Argel) and PV24 (Coscorrón). Although, in both varieties onset of coloration occurred during the seed filling phase, it was desynchronized. While in PV172 the coloration occurs during the middle part of the seed maturation, in PV24 the whitening occurs during the last part of this period and when the seed start to dry (Fig. 1). Both varieties have a similar time to flowering, but PV24 is delayed in pod development which is associated with a shorter seed-filling days of PV172 [23]. It is probably a consequence that PV24 is a bigger seed, which suggest that it requires a longer period for reserves translocation compared to the smaller seed of PV172. Therefore, according to the observation in the Fig. 1, the turn of color in PV172 is shared to different biochemical processes in comparison with PV24 (Figs. 3, 4 and 5).

On the other hand, the higher number of genes shared exclusively in PV172 stages, compared to PV24, is related to a desynchrony between the seed color change and the rest of the processes in the seed development between both varieties (Figs. 1 and 2). The color change in PV172 occurs in a shorter period of time than PV24. Consequently, the transcriptomic changes during these three phases were reduced in the black bean, compared to the white variety. These results suggest that the rapid accumulation of anthocyanin in PV172 may be associated to the precocious behavior of this variety. At the same time, several genes that were expressed in PV172 were not transcriptionally activated in PV24, suggesting that at these stages other processes are correlating with the color change between both varieties (Fig. 3a). The results of the DEGs under the equivalent coloration stages between the varieties show that more synchrony can be found between the C1 stages, while it decreases in the C2 and C3 stages (Fig. 3b). This difference in color change might be explained by phenology desynchrony in the seed development, where in PV24 the dehydration phase starts during C2 and C3 stages, while in PV172 the seeds even gain dry weight.

The results may be a consequence of a broad genetic background, as the Chilean bean collection includes different Mesoamerican and Andean races [35]. Desynchrony of phenological processes is also found in wild or cultivated *Arabidopsis* and bean accessions for flowering time and other traits, because they show a wide range of genetic variation [23, 36, 37].

Different processes related to anthocyanin biosynthesis occur simultaneously and differentially in both varieties. It is also observed on the level of gene ontology and KEGG pathway. Groups of genes belong of KEGG category “starch and sucrose metabolism” and GO MF category “hydrolase activity, hydrolyzing O-glycosyl compounds” were specifically enriched in PV172 (Fig. 4). Some of these genes encode to enzymes that are accumulated in vacuoles or dry seed coat. However, they are required during the seed germination [38–41]. Others genes related with starch and sucrose metabolism, such as encoding to sucrose synthase are important for the reserve accumulation during seed filling [42]. In rice, the gene SCP46 encoding to serine carboxypeptidase regulates seed filling. Both coding genes were enriched in PV172 during the color change, suggesting that the seeds are under filling process.

Similarly, galactosidase activity and galactose metabolism were present in the C3 comparison (Fig. 4). α and β galactosidases were induced in PV172. Galactosidases have been described in seed germination and seed development in other species [43, 44]. α-galactosidase activity reach the higher activity in the mature seed of *Cicer arietum*. This is required for sucrose supply during germination [44].

The KEGG pathway “glycolysis and gluconeogenesis” was differentially induced in PV172 stages compared to PV24 (Fig. 4). Plastid glycolysis is required in sink organs to convert photosynthesis-derived carbohydrates into metabolites that serve as substrates for the biosynthesis of FAs in chloroplasts. One example is the pyruvate kinase, which is induced in PV172, thus providing the pyruvate necessary for the production of plastidial acetyl-CoA [45]. It is also related with the GO “Carboxylic acid biosynthetic process” at the C3 stage. This suggests that PV172 at the C3 stage is involved in a carbon flux from the cytosol to the stroma for fatty acid synthesis (Fig. 4).

The GO analysis showed the enrichment of genes related to stress response and protein association in PV172 (Fig. 4). Among the induced genes in PV172 several small heat shock proteins (sHSPs) were detected, grouped into “response to heat”. They are synthesized in response to high temperature, and other stresses but also during different development stages. HSPs are accumulated from mid-maturation of seeds in *Arabidopsis* and pea, among others [46–48].

In addition, the endoplasmic reticulum (ER) is the site where the protein is folding and disulfide bond formation occur prior to secretion. From the early step of embryo maturation, the ER assembly actively storage proteins for translocation to protein bodies [49]. Genes such as Calreticulin protein, E3 ubiquitin ligase (*RNF5*), ER Oxidoreductin 1 (*ERO1*) and chaperone/heat shock proteins were induced in PV172 (Fig. 4) and enriched in PV172-C1 respect to PV24, suggesting that the synthesis and folding of storage proteins was an active process during the initial phase of the anthocyanin accumulation in the black bean Negro Argel [50].

Polyphenolic compounds in common bean includes different subclasses of flavonoids, highlighting anthocyanins, flavones, flavonols and isoflavonols [51]. Phenolics compound are found in the cotyledon and seed coat, although the higher concentration can be found in the coat. Cinnamic acid 4-hydroxylase (C4H) is the second key enzyme of lignin, flavonoids and hydroxycinnamic acid ester biosynthetic pathway [52]. Defect in *C4H* reduce the flux for production of monolignols and flavonoids [53, 54]. The *C4H_1* gene was highly expressed in PV172 seed coat, although also was expressed in PV24 (Figs. 6 and 7, table S5). Expression changes between both can be explained by the quantity of flavonoids accumulated for each one. PV172 accumulate near to 95 mg/gr DW of total anthocyanin compared with 20 mg/gr DW in other flavonoids in PV24. Therefore, C4H appear to be important to regulate a proportional flux to anthocyanin or other flavonoids in common bean seed.

**Fig. 8:**
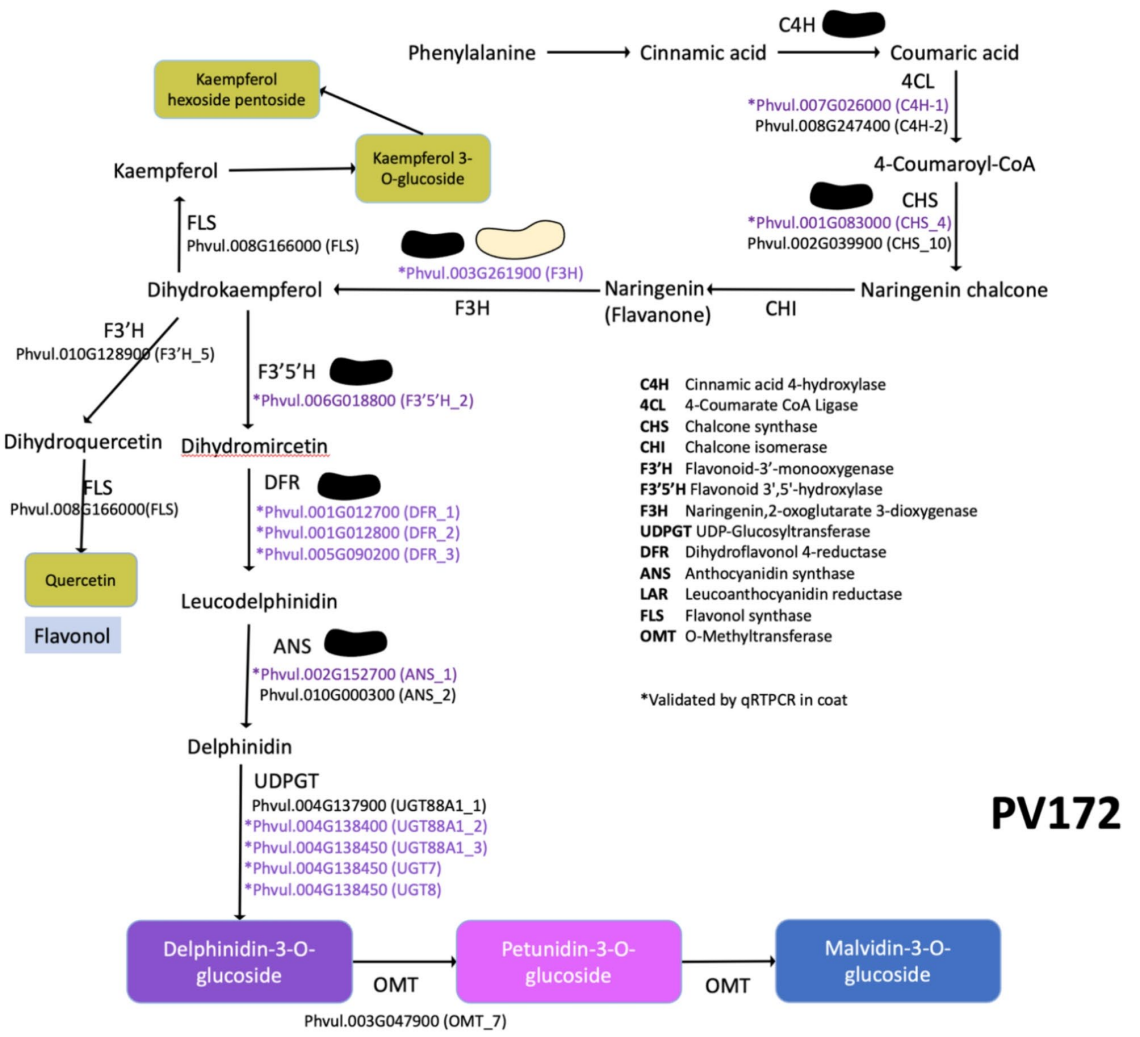
Pathway representation of anthocyanin and others flavonoids in PV172 variety. Genes signed in purple and asterisk have been validated by qRTPCR in coat

The biosynthesis pathway of anthocyanin has been partially described in common bean pods and some genes have been identified coding for their enzymes, such as the genes encoding for *CHS*, *DFR*, *F3H*, *ANS*, *OMT* (XM_007146112, X06411, XM_00716060672, XM_007156079 and XM_007160185.1) [22]. Some of these genes coincided with the induced in PV172 during coloring stages, suggesting that certain similarities can be found between the expression in pod and seed coat genes related with anthocyanin biosynthesis. We identified the genes *C4H_1*, *CHS_4*, *CHS_10*, *DFR1*, *DFR2* and *DFR3* which have not been described previously in common bean anthocyanin biosynthesis (Fig. 7). The CHS is the first enzymes in the flavonoid biosynthesis. In this work, two *CHS* genes were induced in PV172 suggesting the relevance of the first over the last in *P. vulgaris* seeds (Figs. 6 and 7), as well as, has been described as an important enzyme for anthocyanin biosynthesis in *Malus*, eggplant, *Ophiorrhiza japonica*, among others [55–57]. In the flavonoid pathway other key enzyme is F3H, which determine the dihydroflavonol synthesis from flavanone [58]. The inhibition of *F3H* promote the accumulation of Flavan-4-ol. The *F3H* gene was induced in PV172 respect to PV24, suggesting the accumulation of dihydrokaempferol (Figs. 7 and 8). Previously, flavonols such as kaempferol, quercetin, myricetin and their derivatives have been found in common beans varieties [6, 59]. This work report the accumulation of kaempferol glycoside derivative in both varieties; however, quercetin derivates were only found in PV172. Also, the acylation of kaempferol derivatives can be occur in PV24, which is suggested occur by the induction of acyl transferases (*AT1*). Similarly, than anthocyanin, high correlation of quercetin hexoside was found with *UDPGT*, suggesting that this group of genes are important for glycosylation of several types of flavonoids in *P. vulgaris* (Table S5).

In purple common bean pods, the genes *PvF3H* and *PvF3’5’H* direct the biosynthesis of delphinidin based anthocyanin [22]. Recently, a *F3’5’H* was found encoded in the common bean V gene, that in combination to other genes regulate the color expression. This mutation controls the color expression in common bean [60]. The gene *F3’5’H_2* (Phvul.006G018800) was positively regulated in the transcriptome of PV172 and it was induced mainly in the C1 stage (Figs. 6 and 7). Moreover, it was only weakly expressed in PV24. Here, we suggest that additionally to the presence of different variants for this gene, which have been demonstrated by other researchers, it is also transcriptionally regulated during the seed development in the black bean PV172.

Kaempferol and three of its derivatives as well as catechin were found in PV24. White common bean accumulates flavonoids [6, 61]. This is coincided with the induction of genes related with isoflavonoids, flavones and flavonols at the last phase (C3), during the seed dehydration of PV24 (Fig. 5). It suggests that other classes of flavonoids are preferentially accumulated in PV24 during the final phase of seed development.

DFR is considered to be the key enzyme that regulate the direction of carbon flux in anthocyanin pigmentation [62]. The high expression of three different genes (*DFR1*,* DFR2* and *DFR3*) in seed coat of black common bean suggest that it is a very important step for the regulation of anthocyanin synthesis. The *DFR4* gene also have high expression in PV24 (Fig. 6). Here, we suggest that *DFR4* can be involved in the synthesis of leucocyanidin from dihydroquercetin to catechin biosynthesis (Table 1, Fig S3), which can be observed in the HPLC results where PV24 has the major levels of proanthocyanidins. Although, our result showed a high expression of the flavonoid pathway in PV172, both varieties had activity up to the enzyme DFR. Therefore, these results strongly suggest that, the difference in the anthocyanin biosynthesis can be explained by the enzymes involved downstream to the biosynthesis.

The last step for the synthesis of anthocyanidin is catalyzed by ANS. The *ANS_1* gene was the only found in *P. vulgaris* genome. Because the anthocyanidin is unstable, it is glycosylated by UFGTs. The glycosylation is important as signal for transport to vacuole [63, 64]. Four genes coding for UGTs were significative induced in PV172 respect to PV24 (Fig. 7). These four are involved in anthocyanin glycosylation while another probably participate in the glycosylation of others flavonoids.

On the other hand, OMT can further catalyze the conversion of anthocyanidin glycoside to the methylated compounds. The PV172 accumulated delphinidin 3-hexoside as the major anthocyanin, but also malvidin-3-hexoside, and petunidin-3-hexoside were also found (Table 1). Previously, the three anthocyanin hexosides have been found in others varieties of black beans [6, 9, 65–67]. Malvidin di hexosides were reported previously in beans cultivated in China [66]. Acylated anthocyanins and flavonols are also common in beans [9]. In addition, pelargonidin glycosides and diglycosides have been described in black beans [68], however, they were not found in our samples. The gene *PvOMT4* promote the accumulation of malvidin derivative in common bean pod [22]. Here, we suggest that *OMT_7* is involved in the conversion of delphinidin-3-O-glucoside in the petunidin and malvidin glucosides, the most important anthocyanidins synthetized in the black bean.

Structural anthocyanin genes are controlled by the multiprotein MBW complexes where the R2-R3 MYB TF physically interacts with a basic Helix-Loop-Helix (bHLH) TF and a WD40-repeat (WDR) protein [69]. These TFs in *Arabidopsis* include *PAP1*/*MYB75*, *PAP2*/*MYB90*,* GL3/bHLH001*, *EGL3/bHLH002*, *TT8/bHLH042* and *TTG1* [70–73]. Some orthologous of these genes have been identified in *P. vulgaris* [21, 22, 74]. These TFs genes were not expressed or induced in PV172 transcriptome at the stages evaluated on the seeds. Probably, the induction of these genes can be previous to the C1 stage, before the color change start. Also, it is important to mention that these genes were described in pod tissue but not in seed coat [22].

A TF overexpressed during color formation in PV172 and contrasting to PV24 was identified in the present work. The TF belongs to MYB family (*PvMYB26*, Phvul.010G137500). Previously, a TF *MYB26* was described in flower bud of pea that regulate the cis elements in phenylpropanoid gene pathway [75].

Other TF induced in PV172 was from the family of BHLH known as *ALC* which have a high homology with *PIF3* (PHYTOCHROME-INTERACTING FACTOR 3). These TFs have been involved in the regulation of anthocyanin biosynthesis in *Arabidopsis*. Also, PV172 and PV24 maintain during C1, C2 and C3 stages high expression of *bZIP-HY5* TF, which is required by PHY3 for the induction of anthocyanin genes in *Arabidopsis* [76, 77]. Additionally, other authors reported the role of jasmonate in anthocyanin accumulation [78]. Here, we identified a gene coding for a *TIFY_10A* with a potential role in signaling mediated by jasmonic acid with a possible influence in anthocyanin biosynthesis [79]. On the other hand, lysine-specific demethylase JUMONJI (*JMJ*), regulate gene transcription and chromatin structure by changing the methylation status of lysine residues and play an important role in plant growth and development. The Histone H3K9 demethylase *JMJ25* found epigenetically modulates anthocyanin biosynthesis in poplar [80]. TCPs are plant-specific TFs that play important roles in diverse biological processes. The TCP3 interacts with MYB12 or MYB111, thus regulating the expression of flavonoid biosynthetic genes in *Arabidopsis* [81]. Us results showed different TFs such as *TCP_2*, *JUMONJI* or *MYB26* TF, between others, that were induced in PV172. These results suggest that other genes still not identified can control the expression of genes related with anthocyanin biosynthesis (Fig. S2).

## Conclusion

This research provides new insights related with the anthocyanin biosynthesis in common bean. The accumulation of anthocyanin occurs from seed filling in the black variety Negro Argel (PV172) but the color change process is desynchronized with that happen in Coscorrón (PV24). The black variety induced expression of most of pathway genes, from the early *C4H* until several *UFGTs*, under the three development stages evaluated here. The expression of many of these genes was enriched in seed coat, where the anthocyanins are accumulated. Although the multiprotein MBW complexes was not detected, several others TFs were induced under this process, with potential roles in the regulation of anthocyanin pathway.

## Electronic supplementary material

Below is the link to the electronic supplementary material.

Supplementary Material 1

Supplementary Material 2

Supplementary Material 3

Supplementary Material 4

Supplementary Material 5

Supplementary Material 6

Supplementary Material 7

Supplementary Material 8

Supplementary Material 9

## Data Availability

All the data generated or analyzed in this research are included in this published article (and its supplementary information files). The raw RNA-seq data are available in the NCBI repository by the BioProject ID: PRJNA1104640. It can be accessed from http://www.ncbi.nlm.nih.gov/bioproject/1104640.

## Abbreviations

DEG: Differentially expressed gene
KEGG: Kyoto Encyclopedia of Genes and Genomes
MeOH: Methanol
GO: Gene onthology
DAA: Days after anthesis
SDW: Seed dry weight
FDR: False discovery rate
TF: Transcription factor
HSP: Heat shock protein
CHS: Chalcone synthase
DFR: Dihydroflavonol 4-reductase
F3H: Naringenin, 2-oxoglutarate 3-dioxygenase
ANS: Anthocyanidin synthase
OMT: O-Methyltransferase
UGT: UDP glucosyltransferase
C4H: Cinnamic acid 4-hydroxylase
F3’5’H: Flavonoid-3’,5’-hydroxylase
UFGT UDP-glucose: flavonoid-O-glycosyltransferase

## References

[1] Parca F, Koca YO, Unay A. Nutritional and antinutrients of some pulses seed and their effects on human health. Int J Secondary Metabolite. 2018;5(4):331–42.

[2] Porch TG, Cichy K, Wang W, Brick M, Beaver JS, Santana-Morant D, Grusak MA. Nutritional composition and cooking characteristics of tepary bean (Phaseolus acutifolius Gray) in comparison with common bean (Phaseolus vulgaris L). Genet Resour Crop Ev. 2017;64(5):935–53.

[3] Ombra MN, d’Acierno A, Nazzaro F, Riccardi R, Spigno P, Zaccardelli M, Pane C, Maione M, Fratianni F. Phenolic composition and antioxidant and antiproliferative activities of the extracts of twelve Common Bean (Phaseolus vulgaris L.) endemic ecotypes of Southern Italy before and after cooking. Oxid Med Cell Longev. 2016;2016:1398298. 28105248 10.1155/2016/1398298 PMC5220516

[4] Rodríguez Madrera R, Campa Negrillo A, Suárez Valles B, Ferreira Fernández JJ. Phenolic content and antioxidant activity in seeds of Common Bean (Phaseolus vulgaris L). Foods. 2021;10(4):864. 33921060 10.3390/foods10040864 PMC8071416

[5] Bento J, Riceli P, Bassinello P, Brito E, Zocollo G, Caliari M, Soares M. Phenolic and saponin profile in grains of carioca beans during storage. LWT- Food Sci Technol. 2020;139. 10.1016/j.lwt.2020.110599.

[6] Nina N, Theoduloz C, Tapia G, Jimenéz-Aspee F, Márquez K, Schmeda-Hirschmann G. Changes in polyphenol composition, antioxidant capacity and enzyme inhibition in Phaseolus vulgaris L. Sci Hort. 2023; 317.

[7] Ganesan K, Xu B. Polyphenol-Rich Dry Common beans (Phaseolus vulgaris L.) and their health benefits. Int J Mol Sci. 2017;18(11):2331. 29113066 10.3390/ijms18112331 PMC5713300

[8] Nicolás-García M, Perucini-Avendaño M, Jimenez-Martinez C, Perea-Flores MJ, Gómez-Patiño M, Arrieta-Baez D. Dávila Ortiz G. Bean phenolic compound changes during processing: Chemical interactions and identification. J Food Sci. 2021;86(3):643–55. 33586793 10.1111/1750-3841.15632

[9] Yang QQ, Gan RY, Ge YY, Zhang D, Corke H. Polyphenols in Common beans (Phaseolus vulgaris L.): Chemistry, Analysis, and factors affecting composition. Compr Rev Food Sci Food Saf. 2018;17(6):1518–39. 33350144 10.1111/1541-4337.12391

[10] Naing AH, Kim CK. Roles of R2R3-MYB transcription factors in transcriptional regulation of anthocyanin biosynthesis in horticultural plants. Plant Mol Biol. 2018;98:1–18. 30167900 10.1007/s11103-018-0771-4

[11] Yan H, Pei X, Zhang H, Li X, Zhang X, Zhao M, Chiang VL, Sederoff RR, Zhao X. MYB-Mediated regulation of Anthocyanin Biosynthesis. Int J Mol Sci. 2021;18(6):3103. 10.3390/ijms22063103 PMC8002911 33803587

[12] Povero G, Gonzali S, Bassolino L, Mazzucato A, Perata P. Transcriptional analysis in high-anthocyanin tomatoes reveals synergistic effect of aft and atv genes. J Plant Physiol. 2011;168:270–9. 20888667 10.1016/j.jplph.2010.07.022

[13] Lei T, Huang J, Ruan H, Qian W, Fang Z, Gu C, Zhang N, Liang Y, Wang Z, Gao L, Wang Y. Competition between FLS and DFR regulates the distribution of flavonols and proanthocyanidins in Rubus Chingii Hu. Front Plant Sci. 2023;14:1134993. 36968391 10.3389/fpls.2023.1134993 PMC10031046

[14] Zhu Z, Xing S, Zan W, Wang Y, Wu Q, Yu Y. An O-methyltransferase gene, RrCCoAOMT1, participates in the red flower color formation of Rosa rugosa. Sci Hort. 2024;336:113402.

[15] Shi Y, Chen Z, Shen M, Li Q, Wang S, Jiang J, Zeng W. Identification and Functional Verification of the glycosyltransferase gene family involved in Flavonoid synthesis in Rubus Chingii Hu. Plants. 2024;13(10):1390. 38794460 10.3390/plants13101390 PMC11125054

[16] Yang J, Chen Y, Xiao Z, Shen H, Li Y, Wang Y. Multilevel regulation of anthocyanin-promoting R2R3-MYB transcription factors in plants. Front Plant Sci. 2022;13:1008829. 36147236 10.3389/fpls.2022.1008829 PMC9485867

[17] Li S. Transcriptional control of flavonoid biosynthesis: fine-tuning of the MYB-bHLH-WD40 (MBW) complex. Plant Signal Behav. 2014;9(1):e27522. 24393776 10.4161/psb.27522 PMC4091223

[18] Yin X, Zhang Y, Zhang L, Wang B, Zhao Y, Irfan M, Chen L, Feng Y. Regulation of MYB Transcription Factors of Anthocyanin Synthesis in Lily Flowers. Front Plant Sci. 2021;12:761668. 34925411 10.3389/fpls.2021.761668 PMC8672200

[19] Wang C, Ji W, Liu Y, Zhou P, Meng Y, Zhang P, Wen J, Mysore KS, Zhai J, Young ND, Tian Z, Niu L, Lin H. The antagonistic MYB paralogs RH1 and RH2 govern anthocyanin leaf markings in Medicago truncatula. New Phytol. 2021;229(6):3330–44. 33222243 10.1111/nph.17097 PMC7986808

[20] Krylova EA, Mikhailova AS. Regulation of flavonoid biosynthesis in representatives of the tribe Phaseoleae DC. Plant Biotechnol Breed. 2021;4(3):15–25.

[21] McClean PE, Bett KE, Stonehouse R, Lee R, Pflieger S, Moghaddam SM, Geffroy V, Miklas P, Mamidi S. White seed color in common bean (Phaseolus vulgaris) results from convergent evolution in the P (pigment) gene. New Phytol. 2018;219(3):1112–23. 29897103 10.1111/nph.15259

[22] Hu J, Chen G, Zhang Y, Cui B, Yin W, Yu X, Zhu Z, Hu Z. Anthocyanin composition and expression analysis of anthocyanin biosynthetic genes in kidney bean pod. Plant Physiol Biochem. 2015;97:304–12. 26512970 10.1016/j.plaphy.2015.10.019

[23] Tapia G, Méndez J, Inostroza L, Lozano C. Water shortage affects vegetative and Reproductive stages of Common Bean (Phaseolus vulgaris) Chilean landraces, differentially impacting Grain Yield Components. Plants. 2022;11:749. 35336629 10.3390/plants11060749 PMC8948600

[24] Clavijo Michelangeli JA, Bhakta M, Gezan SA, Boote KJ, Vallejos CE. From flower to seed: identifying phenological markers and reliable growth functions to model reproductive development in the common bean (Phaseolus vulgaris L). Plant Cell Environ. 2013;36(11):2046–58. 23586628 10.1111/pce.12114

[25] Li Z, Trick HN. Rapid method for high-quality RNA isolation from seed endosperm containing high levels of starch. Biotechniques. 2005;38(6):872874876. 10.2144/05386BM05 16018547

[26] Andrews S. FastQC: a quality control tool for high throughput sequence data. 2010. Available online at: https://www.bioinformatics.babraham.ac.uk/projects/fastqc/

[27] Kim D, Langmead B, Salzberg SL. HISAT: a fast spliced aligner with low memory requirements. Nat Methods. 2015;12(4):357–60. 25751142 10.1038/nmeth.3317 PMC4655817

[28] Pertea M, Kim D, Pertea GM, Leek JT, Salzberg SL. Transcript-level expression analysis of RNA-seq experiments with HISAT, StringTie and Ballgown. Nat Protocols. 2016;11(9):1650–67. 27560171 10.1038/nprot.2016.095 PMC5032908

[29] Robinson MD, McCarthy DJ, Smyth GK. edgeR: a Bioconductor package for differential expression analysis of digital gene expression data. Bioinformatics. 2010;26(1):139–40. 19910308 10.1093/bioinformatics/btp616 PMC2796818

[30] Dai X, Zhuang Z, Boschiero C, Dong Y, Zhao PX. LegumeIP V3: from models to crops-an integrative gene discovery platform for translational genomics in legumes. Nucleic Acids Res. 2021;49(D1):D1472–9. 33166388 10.1093/nar/gkaa976 PMC7778993

[31] Buchfink B, Reuter K, Drost HG. (2021). Sensitive protein alignments at tree-of-life scale using DIAMOND. Nat. Methods. 2021;18(4):366–368. 10.1038/s41592-021-01101-x PMC8026399 33828273

[32] VanderPlas J, Granger B, Heer J, Moritz D, Wongsuphasawat K, Satyanarayan A, Lees E, Timofeev I, Welsh B, Sievert S. Altair: interactive statistical visualizations for Python. J Open Source Softw. 2018;3(32):1057.

[33] McKinney W. Pandas: a foundational Python library for data analysis and statistics. Python High Perform Sci Comput. 2011;14:1–9.

[34] Urwat U, Ahmad SM, Masi A, Ganai NA, Murtaza I, Khan I, Zargar SM. Fe and Zn stress induced gene expression analysis unraveled mechanisms of mineral homeostasis in common bean (Phaseolus vulgaris L). Sci. 2021;11:24026. 10.1038/s41598-021-03506-2 PMC8674274 34912040

[35] Paredes M, Becerra V, Tay J, Blair M, Bascur G. Selection of a Representative Core Collection from the Chilean Common Bean Germplasm. Chil JAR. 2010;70(1):3–5.

[36] Shindo C, Bernasconi G, Hardtke CS. Natural genetic variation in Arabidopsis: tools, traits and prospects for evolutionary ecology. Ann Bot. 2007;99(6):1043–54. 17259228 10.1093/aob/mcl281 PMC3243570

[37] Miryeganeh M. Synchronization of senescence and desynchronization of flowering in Arabidopsis thaliana. AoB Plants. 2020;12(3):plaa018. 32577195 10.1093/aobpla/plaa018 PMC7299267

[38] Manzoor S, Naveed N, Bolton A, Khan B, Ali A, Simon P, Faiz S. Variation in seed germination and amylase activity of Diverse Carrot [Daucus carota (L.)] germplasm under simulated Drought stress. HortScience. 2023;58:205–14.

[39] Wang H, Zhou X, Liu C, Li W, Guo W. Suppression of *GhGLU19* encoding β-1,3-glucanase promotes seed germination in cotton. BMC Plant Biol. 2022;22:357. 35869418 10.1186/s12870-022-03748-w PMC9308338

[40] Gojto E. Activity of α-d-Galactosidase in long-stored seeds of Vicia hirsuta. Agriculture. 2023;13:1306.

[41] Srivastava G, Kayastha AM. Β-amylase from starchless seeds of Trigonella foenum-graecum and its localization in germinating seeds. PLoS ONE. 2014;9(2):e88697. 24551136 10.1371/journal.pone.0088697 PMC3925156

[42] Berahim Z, Dorairaj D, Saud HM, Ismail MR. Regulation of sucrose synthase and its association with grain filling in spermine-treated rice plant under water deficit. J Plant Interact. 2019;14(1):464–73.

[43] Zhang Qi, Peng Y, Li X, Chen B, Liu J. β-galactosidase is involved in rice seed germination. Seed Sci Technol. 2021;49(3):261–74.

[44] Arunraj R, Skori L, Kumar A, Hickerson NMN, Shoma N, Samuel MV. Spatial regulation of alpha-galactosidase activity and its influence on raffinose family oligosaccharides during seed maturation and germination in Cicer arietinum. Plant Signal Behav. 2020;15(8):1709707. 31906799 10.1080/15592324.2019.1709707 PMC8570745

[45] Baud S, Wuillème S, Dubreucq B, de Almeida A, Vuagnat C, Lepiniec L, Miquel M, Rochat C. Function of plastidial pyruvate kinases in seeds of Arabidopsis thaliana. Plant J. 2007;52(3):405–19. 17892448 10.1111/j.1365-313X.2007.03232.x

[46] Ma W, Guan X, Li J, Pan R, Wang L, Liu F, Ma H, Zhu S, Hu J, Ruan YL, Chen X, Zhang T. Mitochondrial small heat shock protein mediates seed germination via thermal sensing. Proc Natl Acad Sci USA. 2019;116(10):4716–21. 30765516 10.1073/pnas.1815790116 PMC6410843

[47] Zhao H, Jan A, Ohama N, Kidokoro S, Soma F, Koizumi S, Mogami J, Todaka D, Mizoi J, Shinozaki K, Yamaguchi-Shinozaki K. Cytosolic HSC70s repress heat stress tolerance and enhance seed germination under salt stress conditions. Plant Cell Environ. 2021;44(6):1788–801. 33506954 10.1111/pce.14009

[48] Pai-Hsiang Su Hsou-min, Li. Arabidopsis Stromal 70-kD heat shock proteins are essential for Plant Development and important for Thermotolerance of germinating seeds. Plant Physiol. 2008;146(3):1231–41. 18192441 10.1104/pp.107.114496 PMC2259073

[49] Gemmer M, Chaillet ML, van Loenhout J, Cuevas R, Vismpas D, Grollers-Mulderij M, et al. Visualization of translation and protein biogenesis at the ER membrane. Nature. 2023;614:160–7. 36697828 10.1038/s41586-022-05638-5 PMC9892003

[50] Jain A. Seed storage protein, Functional Diversity and Association with Allergy. Allergies. 2023;3(1):25–38.

[51] Nayak B, Liu RH, Tang J. Effect of processing on phenolic antioxidants of fruits, vegetables, and grains—A review. Crit Rev Food Sci Nutr. 2015;55(7):887–918. 24915381 10.1080/10408398.2011.654142

[52] Blount JW, Korth KL, Masoud SA, Rasmussen S, Lamb C, Dixon RA. Altering expression of cinnamic acid 4-hydroxylase in transgenic plants provides evidence for a feedback loop at the entry point into the phenylpropanoid pathway. Plant Physiol. 2000;122(1):107–16. 10631254 10.1104/pp.122.1.107 PMC58849

[53] Schilmiller AL, Stout J, Weng JK, Humphreys J, Ruegger MO, Chapple C. Mutations in the cinnamate 4-hydroxylase gene impact metabolism, growth and development in Arabidopsis. Plant J. 2009;60(5):771–82. 19682296 10.1111/j.1365-313X.2009.03996.x

[54] Kumar R, Vashisth D, Misra A, Akhtar MQ, Jalil SU, Shanker K, Gupta MM, Rout PK, Gupta AK, Shasany AK. RNAi down-regulation of cinnamate-4-hydroxylase increases artemisinin biosynthesis in Artemisia annua. Sci Rep. 2016;6:26458. 27220407 10.1038/srep26458 PMC4879530

[55] Wu X, Zhang S, Liu X, Shang J, Zhang A, Zhu Z, Zha D. Chalcone synthase (CHS) family members analysis from eggplant (Solanum melongena L.) in the flavonoid biosynthetic pathway and expression patterns in response to heat stress. PLoS ONE. 2020;15(4):e0226537. 32302307 10.1371/journal.pone.0226537 PMC7164647

[56] Tai D, Tian J, Zhang J, Song T, Yao Y. A Malus crabapple chalcone synthase gene, McCHS, regulates red petal color and flavonoid biosynthesis. PLoS ONE. 2014;9(10):e110570. 25357207 10.1371/journal.pone.0110570 PMC4214706

[57] Sun W, Shen H, Xu H, Tang X, Tang M, Ju Z, Yi Y. Chalcone Isomerase a key enzyme for anthocyanin biosynthesis in Ophiorrhiza Japonica. Front Plant Sci. 2019;10:865. 31338101 10.3389/fpls.2019.00865 PMC6629912

[58] Busche M, Acatay C, Martens S, Weisshaar B, Stracke R. Functional characterisation of Banana (Musaspp.) 2-Oxoglutarate-dependent dioxygenases involved in Flavonoid Biosynthesis. Front. Plant Sci. 2021;12:701780. 10.3389/fpls.2021.701780 PMC8415913 34484266

[59] Dueñas M, Sarmento T, Aguilera Y, Benítez V, Molla E, Esteban RM, Martín-Cabrejas MA. Impact of cooking and germination on phenolic composition and dietary fibre fractions in dark beans (Phaseolus vulgaris L.) and lentils (Lens culinaris L). LWT-Food Sci Technol. 2016;66:72–8.

[60] McClean PE, Lee R, Howe K, Osborne C, Grimwood J, Levy S, Haugrud AP, Plott C, Robinson M, Skiba RM, Tanha T, Zamani M, Thannhauser TW, Glahn RP, Schmutz J, Osorno JM, Miklas PN. The Common Bean *V* Gene encodes flavonoid 3’5’ hydroxylase: a major mutational target for flavonoid diversity in Angiosperms. Front Plant Sci. 2022;31(13):869582. 10.3389/fpls.2022.869582 PMC9009181 35432409

[61] Laparra JM, Glahn RP, Miller DD. Bioaccessibility of phenols in common beans (Phaseolus vulgaris L.) and iron (Fe) availability to Caco-2 cells. J Agric Food Chem. 2008;56(22):10999–1005. 18983154 10.1021/jf802537t

[62] Wang X, Chen X, Luo S, Ma W, Li N, Zhang W, Tikunov Y, Xuan S, Zhao J, Wang Y, Zheng G, Yu P, Bai Y, Bovy A, Shen S. Discovery of a DFR gene that controls anthocyanin accumulation in the spiny Solanum group: roles of a natural promoter variant and alternative splicing. Plant J. 2022;111(4):1096–109. 35749258 10.1111/tpj.15877

[63] Buhrman K, Aravena-Calvo J, Ross Zaulich C, Hinz K, Laursen T. Anthocyanic Vacuolar inclusions: from biosynthesis to Storage and possible applications. Front Chem. 2022;10:913324. 35836677 10.3389/fchem.2022.913324 PMC9273883

[64] Yonekura-Sakakibara K, Nakayama T, Yamazaki M, Saito K, Gould K, Davies K, Winefield C, Editors. New York: Springer; 2009. 169–85.

[65] Choung MG, Choi BR, An YN, Chu YH, Cho YS. Anthocyanin profile of Korean cultivated kidney bean (Phaseolus vulgaris L). J Agric Food Chem. 2003;51(24):7040–3. 14611168 10.1021/jf0304021

[66] Lin LZ, Harnly JM, Pastor-Corrales MS, Luthria DL. The polyphenolic profiles of common bean (Phaseolus vulgaris L). Food Chem. 2008;107(1):399–410. 25544796 10.1016/j.foodchem.2007.08.038 PMC4276374

[67] Xu B, Chang SK. Total phenolic, phenolic acid, anthocyanin, flavan-3-ol, and flavonol profiles and antioxidant properties of pinto and black beans (Phaseolus vulgaris L.) as affected by thermal processing. J Agric Food Chem. 2009;57(11):4754–64. 19492791 10.1021/jf900695s

[68] Kan L, Nie S, Hu J, Liu Z, Xie M. Antioxidant activities and anthocyanins composition of seed coats from twenty-six kidney bean cultivars. J Func Foods. 2016;2:622–31.

[69] Xu W, Dubos C, Lepiniec L. Transcriptional control of flavonoid biosynthesis by MYB-bHLH-WDR complexes. Trends Plant Sci. 2015;20(3):176–85. 25577424 10.1016/j.tplants.2014.12.001

[70] Wei Z, Cheng Y, Zhou C, Li D, Gao X, Zhang S, Chen M. Genome-wide identification of direct targets of the TTG1-bHLH-MYB complex in regulating trichome formation and Flavonoid Accumulation in Arabidopsis Thaliana. Int J Mol Sci. 2019;20(20):5014. 31658678 10.3390/ijms20205014 PMC6829465

[71] Baudry A, Caboche M, Lepiniec L. TT8 controls its own expression in a feedback regulation involving TTG1 and homologous MYB and bHLH factors, allowing a strong and cell-specific accumulation of flavonoids in Arabidopsis thaliana. Plant J. 2006;46(5):768–79. 16709193 10.1111/j.1365-313X.2006.02733.x

[72] Feyissa DN, Lovdal T, Olsen KM, Slimestad R, Lillo C. The endogenous GL3, but not EGL3, gene is necessary for anthocyanin accumulation as induced by nitrogen depletion in Arabidopsis rosette stage leaves. Planta. 2009;230(4):747–54. 19621239 10.1007/s00425-009-0978-3

[73] Li SF, Allen PJ, Napoli RS, Browne RG, Pham H, Parish RW. MYB-bHLH-TTG1 regulates Arabidopsis seed Coat Biosynthesis pathways directly and indirectly via multiple tiers of transcription factors. Plant Cell Physiol. 2020;61(5):1005–18. 32154880 10.1093/pcp/pcaa027

[74] Kavas M, Abdulla MF, Mostafa K, Seçgin Z, Yerlikaya BA, Otur Ç, Gökdemir G, Kurt Kızıldog ˘an A, Al-Khayri JM, Jain SM. Investigation and expression analysis of R2R3-MYBs and anthocyanin biosynthesis-related genes during seed Color Development of Common Bean (Phaseolus vulgaris). Plants. 2022;11(23):3386. 36501424 10.3390/plants11233386 PMC9736660

[75] Yan H, Pei X, Zhang H, Li X, Zhang X, Zhao M, Chiang VL, Sederoff RR, Zhao X. MYB-Mediated regulation of Anthocyanin Biosynthesis. Int J Mol Sci. 2021;22(6):3103. 33803587 10.3390/ijms22063103 PMC8002911

[76] Bernal-Gallardo JJ, Zuñiga-Mayo VM, Marsch-Martinez N, de Folter S. Novel roles of SPATULA in the Control of Stomata and Trichome Number, and anthocyanin biosynthesis. Plants. 2023;12(3):596. 36771679 10.3390/plants12030596 PMC9919660

[77] Ma Z, Wei C, Cheng Y, Shang Z, Guo X, Guan J. RNA-Seq analysis identifies transcription factors involved in anthocyanin biosynthesis of ‘Red Zaosu’ Pear Peel and Functional Study of PpPIF8. Int J Mol Sci. 2022;23(9):4798. 35563188 10.3390/ijms23094798 PMC9099880

[78] Qi T, Song S, Ren Q, Wu D, Huang H, Chen Y, Fan M, Peng W, Ren C, Xie D. The Jasmonate-ZIM-domain proteins interact with the WD-Repeat/bHLH/MYB complexes to regulate Jasmonate-mediated anthocyanin accumulation and trichome initiation in Arabidopsis thaliana. Plant Cell. 2011;23(5):1795–814. 21551388 10.1105/tpc.111.083261 PMC3123955

[79] Zhu D, Li R, Liu X, Sun M, Wu J, Zhang N, Zhu Y. The positive Regulatory roles of the TIFY10 proteins in plant responses to alkaline stress. PLoS ONE. 2014;9(11):e111984. 25375909 10.1371/journal.pone.0111984 PMC4222965

[80] Fan D, Wang X, Tang X, Ye X, Ren S, Wang D, Luo K. Histone H3K9 demethylase JMJ25 epigenetically modulates anthocyanin biosynthesis in poplar. Plant J. 2018;96(6):1121–36. 30218582 10.1111/tpj.14092

[81] Li S, Zachgo S. TCP3 interacts with R2R3-MYB proteins, promotes flavonoid biosynthesis and negatively regulates the auxin response in Arabidopsis thaliana. Plant J. 2013;76(6):901–13. 24118612 10.1111/tpj.12348

