# Supplementary material for: Transcriptome analysis reveals biosynthesis and regulation of flavonoid in common bean seeds during grain filling
Source: BMC Plant Biol. 2024 Oct 1;24:916. doi: 10.1186/s12870-024-05593-5 (PMC11443926; doi:10.1186/s12870-024-05593-5)
Supplement: Supplementary file 4 — Supplementary Material 4 [file 12870_2024_5593_MOESM4_ESM.docx]

Table S4: HPLC-DAD-ESI-MS analysis of the main compounds in the SMEE of Chilean bean landraces under optimal watering. Detection in the negative ion mode for flavonols, catechins, procyanidins and ferulic acid. Detection in the positive ion mode for anthocyanins.

| **Rt**  **(min)** | **[M-H]^-^/[M+H]^+^**  **detected** | **Molecular formula** | **Error (ppm)** | **λ_max_**  **(nm)** | **MS**  **fragmentation** | **Identification/tentative identification** |
| --- | --- | --- | --- | --- | --- | --- |
| 10.4 | 451.1250 | C_21_H_24_O_11_ | -0.9 | 277 | 289.0722 (100), 137.0248 (11) | Catechin hexoside |
| 12.4 | 577.1342 | C_30_H_25_O_12_ | 0.7 | 276 | 425.0871 (67), 407.0758 (69), 289.0711 (100), 245.0797 (27), 161.0245 (29), 137.0235 (25), 125.0237 (78) | Procyanidin dimer B |
| 14.5 | 865.1996 | C_45_H_37_O_17_ | -1.8 | 276 | 577.1314 (13), 407.0810 (33), 289.0715 (63), 245.0424 (26), 125.0235 (45) | Procyanidin trimer B |
| 14.9 | 289.0720 | C_15_H_13_O_6_^-^ | -0.7 | 275 | 245.0822 (84), 205.0508 (35), 179.0353 (26), 137.0247 (40), 123.0463 (52), 109.0305 (56) | Catechin* |
| 17.6 | 595.1323 | C_26_H_28_O_16_ | -3.0 | 354, 268 | 301.0340 (100) | Quercetin hexoside pentoside |
| 19.4 | 579.1380 | C_26_H_28_O_15_ | 4.3 | 347, 267 | 285.0410 (100), 255.0307(27) | Kaempferol hexoside pentoside |
| 21.9 | 447.0935 | C_21_H_20_O_11_ | 0.4 | 346, 265 | 285.0412 (49), 255.0296 (96), 227.0344 (100) | Kaempferol 3-*O*-glucoside* |
| 23.2 | 193.0512 | C_10_H_10_O_4_ | 3.1 | 322, 217 | 178.0281 (43), 134.0374 (100) | Ferulic acid* |
| 23.4 | 489.1036 | C_23_H_22_O_12_ | -0.4 | 347, 265 | 395.0942 (7), 285.0412 (92), 255.0302 (100), 227.0341 (36), 151.0043 (10) | Kaempferol acetyl hexoside |
| 35.8 | 285.0417 | C_15_H_10_O_6_ | 4.2 | 366 | 285.0417 (100) | Kaempferol* |
| Anthocyanins | |  |  |  |  |  |
| 14.4 | 655.1863 | C_29_H_35_O_17_ | 0.66 | 520 | 493.1342 (39), 331.0811 (100) | Malvidin dihexoside |
| 14.8 | 465.1024 | C_21_H_21_O_12_ | -0.06 | 524 | 303.0499 (100) | Delphinidin 3-*O*-hexoside |
| 16.9 | 479.1182 | C_22_H_23_O_12_ | -0.21 | 526 | 317.0656 (100) | Petunidin 3-*O*-hexoside |
| 18.5 | 493.1342 | C_23_H_25_O_12_ | 0.14 | 516 | 331.0811 (100) | Malvidin 3-*O*-hexoside 1 |
| 19.5 | 493.1344 | C_23_H_25_O_12_ | 0.35 | 516 | 331.0811 (100) | Malvidin 3-*O*-hexoside 2 |
| 21.2 | 551.1028 | C_24_H_23_O_15_ | -0.31 | 520 | 303.0500 (100) | Delphinidin 3-(6’’-malonylhexoside) |

*Identified by comparison with standards.
