# Supplementary figures and images for: Transcriptome analysis reveals biosynthesis and regulation of flavonoid in common bean seeds during grain filling
Source: BMC Plant Biol. 2024 Oct 1;24:916. doi: 10.1186/s12870-024-05593-5 (PMC11443926; doi:10.1186/s12870-024-05593-5)

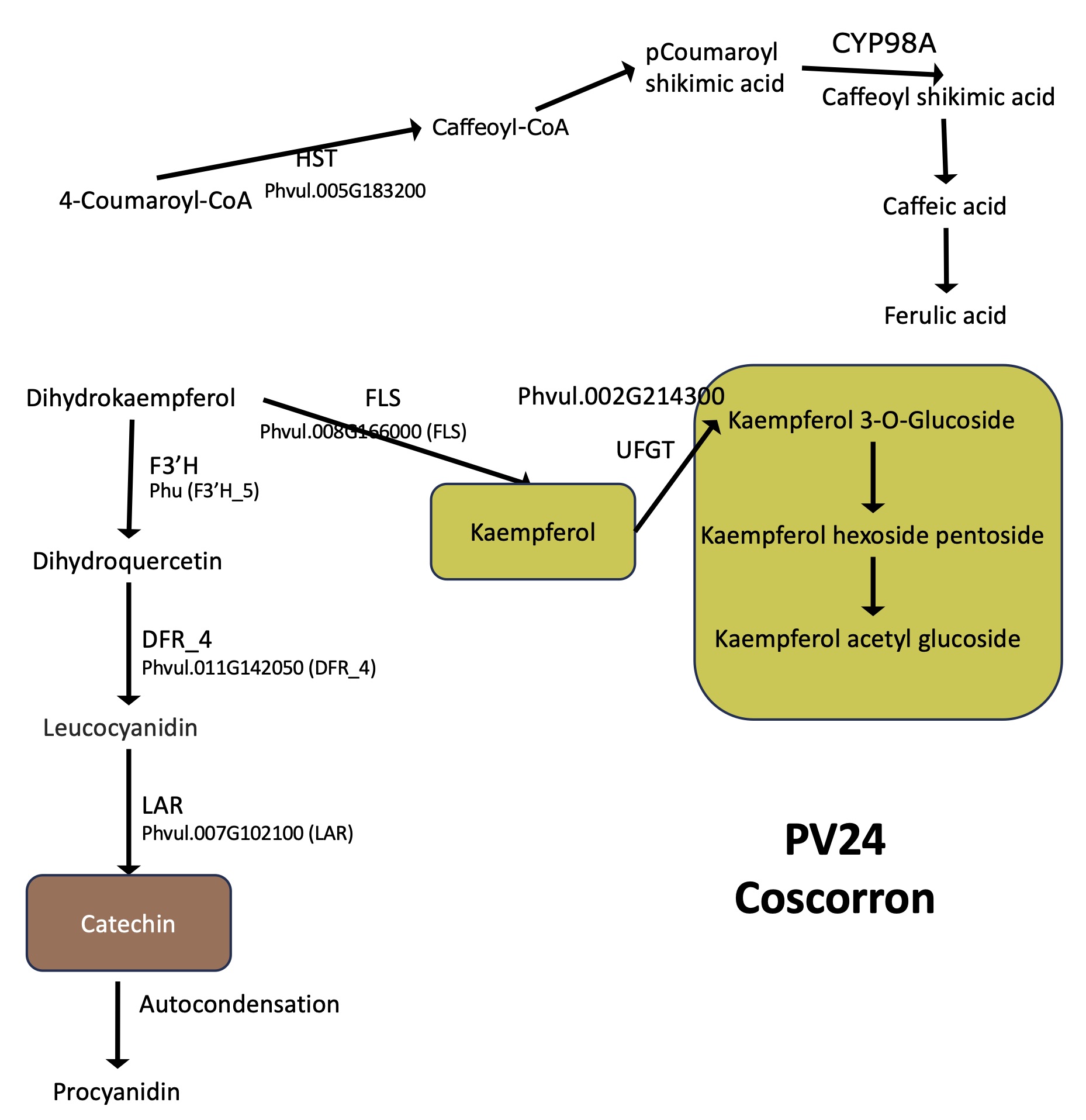

Supplement: Supplementary file 6 — Supplementary Material 6 [file 12870_2024_5593_MOESM6_ESM.jpg]

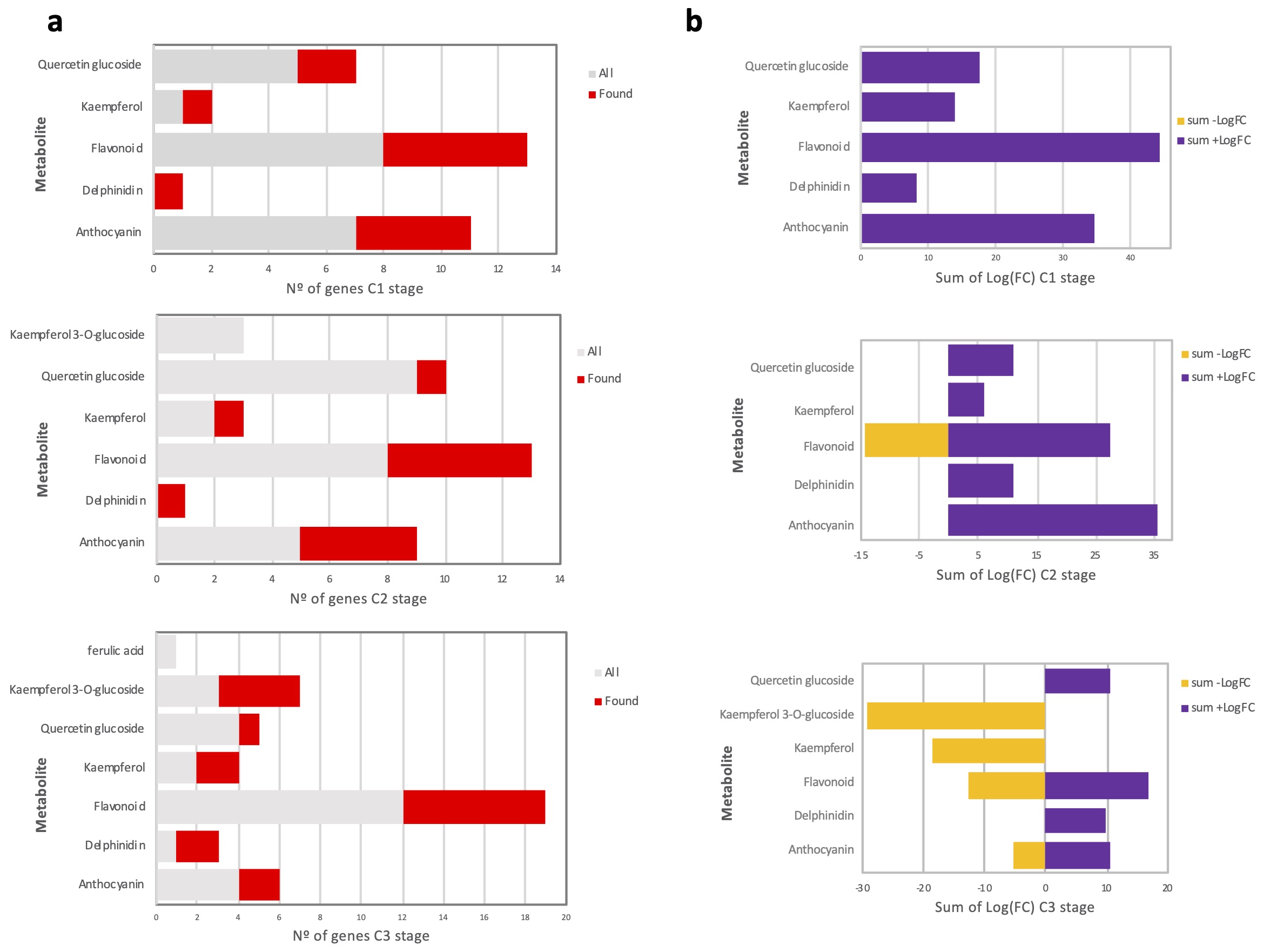

Supplement: Supplementary file 7 — Supplementary Material 7 [file 12870_2024_5593_MOESM7_ESM.jpg]

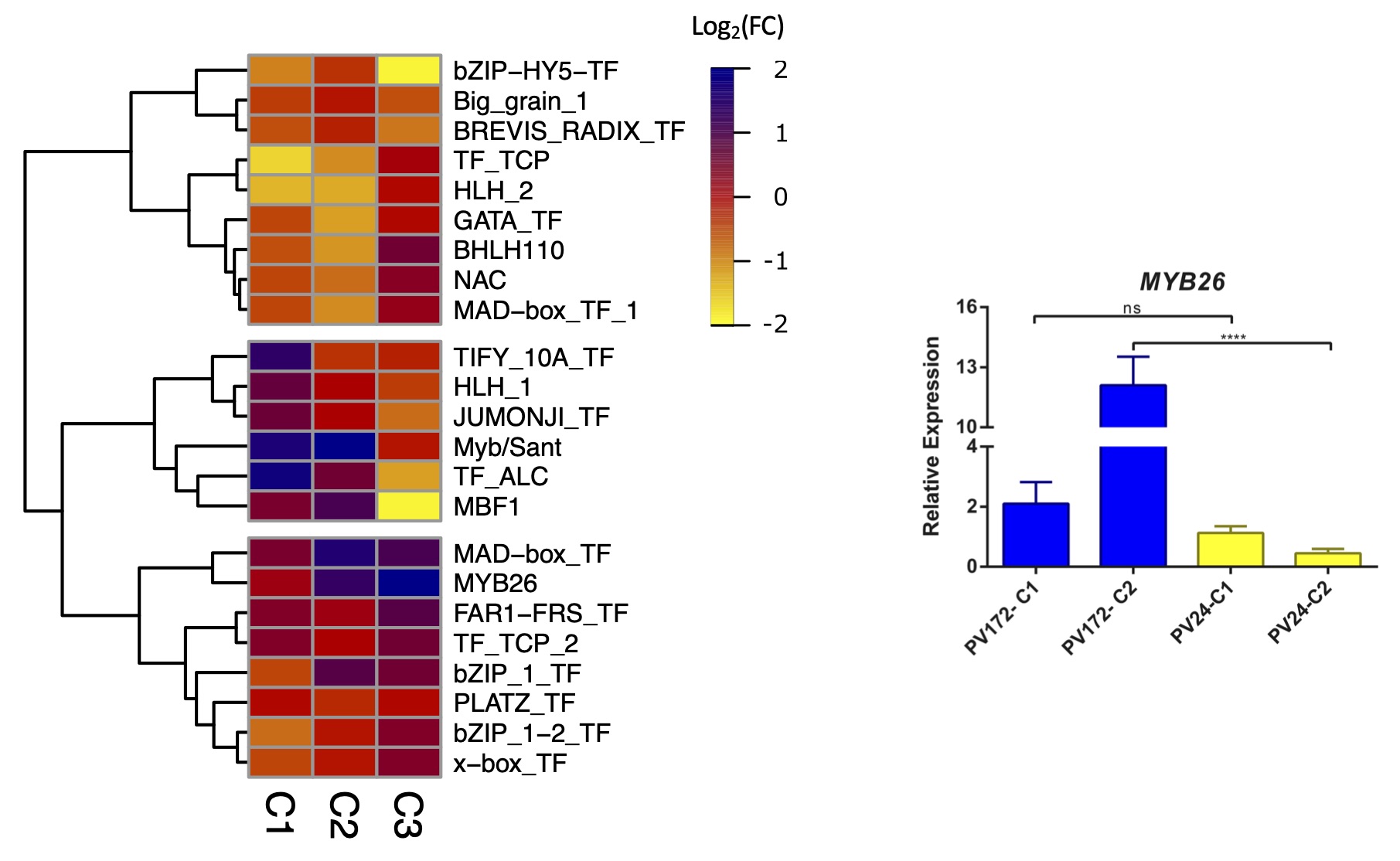

Supplement: Supplementary file 8 — Supplementary Material 8 [file 12870_2024_5593_MOESM8_ESM.jpg]

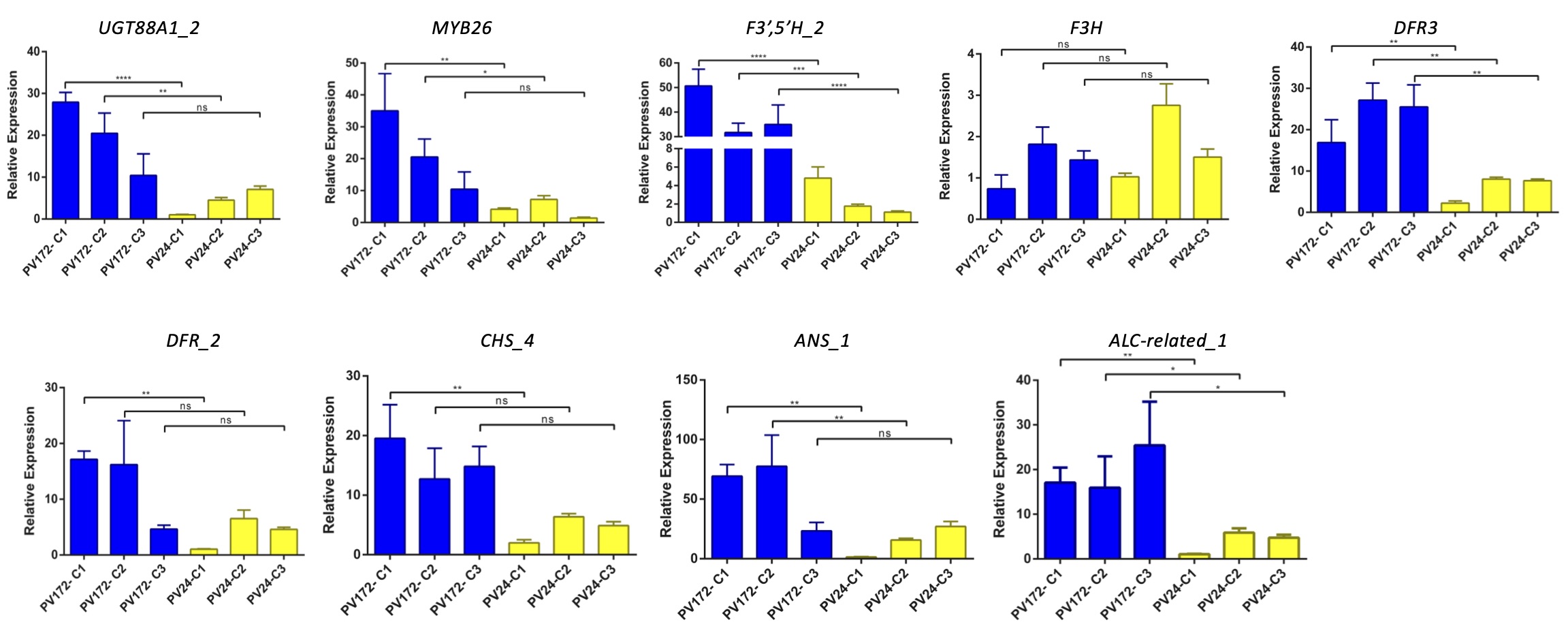

Supplement: Supplementary file 9 — Supplementary Material 9 [file 12870_2024_5593_MOESM9_ESM.jpg]
